# Switching Reactive Oxygen Species into Reactive Nitrogen Species by Photocleaved O_2_‐Released Nanoplatforms Favors Hypoxic Tumor Repression

**DOI:** 10.1002/advs.202101065

**Published:** 2021-08-08

**Authors:** Tao Luo, Duo Wang, Lidong Liu, Yan Zhang, Chuangye Han, Ying Xie, Yan Liu, Jingchen Liang, Guanhua Qiu, Hongxue Li, Danke Su, Junjie Liu, Kun Zhang

**Affiliations:** ^1^ Department of Gastrointestinal Surgery Department of Medical Ultrasound Department of Radiology and The Fifth Department of Chemotherapy Guangxi Medical University Cancer Hospital Guangxi Medical University 71 Hedi Road Nanning 530021 P. R. China; ^2^ Department of Medical Ultrasound and Central Laboratory and Ultrasound Research and Education Institute Shanghai Tenth People's Hospital Shanghai Engineering Research Center of Ultrasound Diagnosis and Treatment Tongji University School of Medicine 301 Yan‐chang‐zhong Road Shanghai 200072 P. R. China; ^3^ Department of Hepatobiliary Surgery The First Affiliated Hospital of Guangxi Medical University Guangxi Medical University 6 Shuangyong Road Nanning 530021 P. R. China; ^4^ Life Science Institute Guangxi Medical University 22 Shuangyong Road Nanning 530021 P. R. China

**Keywords:** hypoxia mitigation, PDE5 inhibition, photocleaved O_2_ release, reactive nitriogen species, reactive oxygen species, short lifetime

## Abstract

In various reactive oxygen species (ROS)‐based antitumor approaches (e.g., photodynamic therapy), increasing attentions are made to improve ROS level, but the short lifetime that is another decisive hurdle of ROS‐based antitumor outcomes is not even explored yet. To address it, a photocleaved O_2_‐released nanoplatform is constructed to release and switch ROS into reactive nitrogen species (RNS) for repressing hypoxic breast tumor. Systematic explorations validate that the nanoplatforms can attain continuous photocontrolled O_2_ release, alleviate hypoxia, and elevate ROS level. More significantly, the entrapped PDE5 inhibitor (PDE5‐i) in this nanoplatform can be enzymatically decomposed into nitric oxide that further combines with ROS to generate RNS, enabling the persistent antitumor effect since RNS features longer lifetime than ROS. Intriguingly, ROS conversion into RNS can help ROS to evade the hypoxia‐induced resistance to ROS‐based antitumor. Eventually, RNS production unlocks robust antitumor performances along with ROS elevation and hypoxia mitigation. Moreover, this extraordinary conversion from ROS into RNS also can act as a general method to solve the short lifetime of ROS.

## Graphical abstract

An unprecedented solution to address the short lifetime of reactive oxygen species (ROS), i.e., switching short‐lived ROS into long‐lived reactive nitrogen species (RNS), is established and enabled by a photocleaved O_2_‐released nanoplatform. It combines with O_2_ release‐enhanced ROS/RNS production and hypoxia alleviation to cooperatively favor the persistent hypoxic tumor repression.

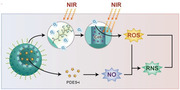

## 1 Introduction

Depending on the safe, targeting, and precise properties, various reactive oxygen species (ROS)‐based antitumor strategies have been developed and gained increasing interests, e.g., sonodynamic therapy (SDT), photodynamic therapy (PDT), chemodynamic therapy (CDT), and fenton or fenton‐like nanocatalytic medicine, etc.^[^ ^1^ ^]^ Great efforts have been devoted to improve ROS production since the treatment outcome closely correlates with ROS level,^[^ ^2^ ^]^ e.g., tumor microenvironment modulation, hypoxia alleviation, nonstoichiometric metal oxides for inherent electron–hole pairs’ separation, external stimuli‐triggered electron–hole pairs’ separation, and continuous cavitation.^[^ ^3^ ^]^ As well, some auxiliary means were integrated with ROS‐based oncolytic strategies for magnifying the antitumor performances.^[^ ^3c,i^ ^]^ Despite improving ROS level to some extent, these ROS‐based antitumor approaches still suffer from short action time due to the inherent short lifetime (SLT) of ROS,^[^ ^4^ ^]^ e.g., 10^−6^ s for singlet oxygen (^1^O_2_) and 10^−9^ s for hydroxyl free radical (·OH),^[^ ^5^ ^]^ which severely impaired ROS‐based therapeutic outcomes.^[^ ^4a^ ^]^ This phenomenon can be attributed to that fact that the tough SLT barrier inevitably results in the failures of oxidizing and destroying DNA since ^1^O_2_ will rapidly annihilate before it can reach and enter nuclei or mitochondria. Unfortunately, no available solution in current ROS‐based antitumor methods has not been found yet; and even no attempts have been made to address this concern.

**Figure 1:**
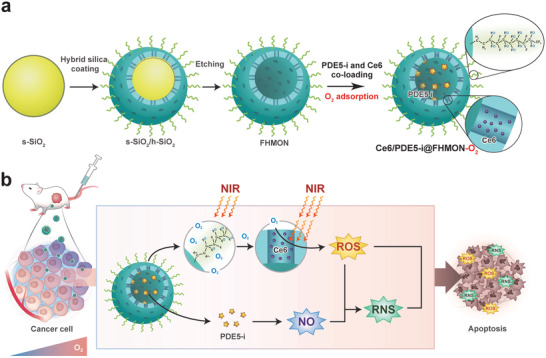
Schematic illustration on the synthetic procedures of such a photocleaved O_2_‐released nanoplatform (i.e., Ce6/PDE5‐i@FHMON‐O_2_) and the switching from ROS to RNS. a) Detailed synthesis process of Ce6/PDE5‐i@FHMON‐O_2_ and its intermediate products such as FHMON, Ce6@FHMON, and Ce6/PDE5‐i@FHMON. b) The schematics for illustrating the underlying principle of RNS production arising from ROS fusion with NO and elucidating the activated ROS/RNS pathway against tumors.

To address this hurdle, a photocleaved O_2_‐released nanoplatform (Ce6/PDE5‐i@FHMON‐O_2_) has been constructed to produce and switch ROS into reactive nitrogen species (RNS) for repressing hypoxic MCF‐7 breast tumor, wherein fluorocarbon‐chelated hollow mesoporous organosilica nanoparticles (FHMONs) act as supports to coload phosphodiesterase‐5 inhibitors (PDE5‐i) and Ce6 photosensitizers and bind with O_2_ (**Figure** **1a**). Herein, the entrapped PDE5‐i can activate cGMP/nitric oxide (NO) pathway to produce NO,^[^ ^6^ ^]^ and NO further combined with ROS (i.e., ^1^O_2_) to give birth to RNS (e.g., ONOO—) (Figure 1b).^[^ ^7^ ^]^ Inspired by the fact that RNS features higher stability, longer lifetime (10^−2^ s) and more persistent antitumor performance than ROS,^[^ ^4^, ^5^ ^]^ the continuous antitumor action mediated by RNS is within easy reach. This unprecedented conversion from ROS to RNS is highly desirable for resolving the SLT hurdle and exerting long‐term antitumor effects.

Depending on the high affinity of fluorocarbon chains with O_2_,^[^ ^8^ ^]^ this nanoplatform can absorb and release O_2_ in response to near infrared (NIR) light irradiation. Akin to previous cases where O_2_ release enabled hypoxia mitigation and ROS elevation,^[^ ^9^ ^]^ the adequate O_2_ supply from this photocleaved O_2_‐released nanoplatform is also expected to alleviate the ubiquitous hypoxic microenvironment and augment ROS production. It will favor more conversion of ROS into RNS since RNS birth derives from ROS fusion with NO, which, thus, further benefit the activations of ROS/RNS pathways for highly efficient PDT against hypoxic breast cancer. Notably, the switching of ROS into RNS can help ROS to evade the hypoxia‐induced resistance to PDT, magnifying the antitumor outcomes. More significantly, this pioneering work and its underlying principle open up a new direction in ROS‐based antitumor methods (e.g., CDT, SDT, PDT, etc.), which means that this unprecedented conversion strategy from ROS into RNS can act as a general method to resolve SLT and will arouse an SLT‐associated research upsurge.

## 2 Results and Discussion

### 2.1 Synthesis of Photocleaved O_2_‐Released Nanoplatform (i.e., Ce6/PDE5‐i@FHMON‐O_2_)

**Figure 2:**
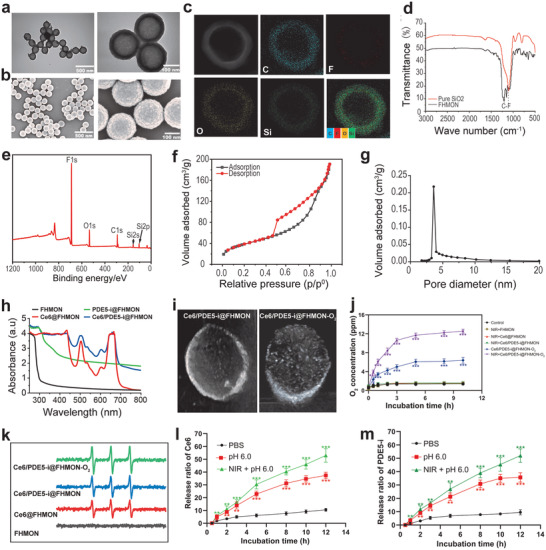
Synthesis and characterizations of Ce6/PDE5‐i@FHMON‐O_2_ and its merits in oxygen release and ROS production. a Transmission electron microscope (TEM) and b) scanning electron microscope (SEM) images of FHMON carriers. c) Atomic mapping images of FHMON carriers to monitor distributions of each atom, wherein fluorine atoms are uniformly distributed in shells of FHMONs. d) FTIR and e) XPS spectra of FHMON carriers, wherein the presence of fluorocarbon chains is also confirmed. (f N_2_ adsorption–desorption isotherm and g) pore diameter distribution profile of FHMON carriers. h) UV–vis spectra of different samples including FHMON, Ce6@FHMON, PDE5‐i@FHMON, and Ce6/PDE5‐i@FHMON, which suggest the successful loading and coloading of Ce6 and PDE5‐i. i) Ultrasonic images of Ce6/PDE5‐i@FHMON and Ce6/PDE5‐i@FHMON‐O_2_ (dose: 2 mg mL^−1^ FHMON) after 660 nm laser irradiation, demonstrating O_2_ adsorption and release from Ce6/PDE5‐i@FHMON‐O_2_. j) Time‐dependent O_2_ concentration variations of different treatments (e.g., control, NIR + FHMON, NIR + Ce6@FHMON, NIR + Ce6/PDE5‐i@FHMON, Ce6/PDE5‐i@FHMON‐O_2_, and NIR + Ce6/PDE5‐i@ FHMON‐O_2_). k) Electron spin resonance spectra of different samples (e.g., FHMON, Ce6@FHMON, Ce6/PDE5‐i@FHMON, and Ce6/PDE5‐i@FHMON‐O_2_) in the presence of 660 nm laser irradiation, wherein DMPO was used as ^1^O_2_ capturing agent for recognizing ^1^O_2_. Release profiles of l) Ce6 and m) PDE5‐i from Ce6/PDE5‐i@FHMON‐O_2_ as a function of incubation time. Note, the parameters of 660 nm laser were set as power density: 0.1 W cm^−2^, duration time: 1 min per irradiation, cycle: 5, and interval time: 1 min between two irradiations. Data are presented as the mean ± SD (*n* = 3). Statistical analyses were performed using a Student's *t*‐test in Prism software, and *P < 0.05, **P < 0.01, and ***P < 0.001, respectively.

FHMON carriers were easily accessible according to the classic hydrophobic layer‐protected etching method (Figure 1a).^[^ ^8b^ ^]^ TEM and SEM images show the uniform distribution of FHMONs (**Figure** **2a**,b). The determined particle size (190 nm) is approximately identical to that via dynamic light scattering analysis (Figure S1, Supporting Information). Atom mapping, Fourier transform infrared spectroscopy (FTIR) spectra and wide‐angle X‐ray photoelectron spectroscopy (XPS) spectra reveal the presence of evenly distributed F atoms in shells, meaning the successful insertion of fluorocarbon chains during FHMON synthesis (Figure 2c–e). Beside SEM observation, the porous surface is also demonstrated by N_2_ adsorption–desorption isotherms and pore diameter distributions, which determine that FHMON carriers are available for the coloading of Ce6 and PDE5‐i (Figure 2f,g). Typical characteristic UV–vis peaks of PDE5‐i and Ce6 are found in Ce6/PDE5‐i@FHMON‐O_2_ (Figure 2h), demonstrating the successful coloading of PDE5‐i and Ce6. As well, the progressive increase of zeta potential also reflects Ce6/PDE5‐i entrapments and O_2_ adsorption by FHMONs in sequence when comparing Ce6/PDE5‐i@FHMON‐O_2_ and its intermediates (Figure S2, Supporting Information), during which particle size fails to vary (Figure S1, Supporting Information).

By virtue of the high miscibility between fluorocarbon molecules with oxygen, O_2_ is expected to be tethered to fluorocarbon chains in FHMONs. Herein, ultrasound contrast imaging technology whose aim is set to detect bubbles was used to explore the adsorption and release of O_2_ by FHMON, and the loading amount of O_2_ in Ce6/PDE5‐i@FHMON‐O_2_ is determined to be 0.68%. The enhanced ultrasound contrast and massive white dots‐represented bubbles evidenced O_2_ bubbles birth (Figure 2i). Intriguingly, the released O_2_ bubbles from Ce6/PDE5‐i@FHMON‐O_2_ almost illuminate the container in comparison to Ce6/PDE5‐i@FHMON in the presence of 660 nm laser irradiation. This phenomenon adequately demonstrates the successful O_2_ binding to fluorocarbon chains and on‐demand O_2_ release. More significantly, the release profiles of O_2_ from Ce6/PDE5‐i@FHMON‐O_2_ were monitored. Despite spontaneous O_2_ dissociation to some extent, NIR irradiation‐induced mild heat (below 42 °C, Figure S3, Supporting Information) further triggers a considerably elevated O_2_ release (Figure 2j). In light of the positive correlation between oxygen content and ROS level, the sufficient O_2_ supply in this nanoplatform favors massive ROS production (Figure 2k). Meanwhile, FHMON could maintain good stability in phosphate buffered solution and fetal bovine serum (Figure S4, Supporting Information). The loading proportions of PDE5‐i and Ce6 in Ce6/PDE5‐i@FHMON‐O_2_ are determined to be 4.21% and 4.07%, respectively, which were obtained according to their standard curves of concentration relating to their characteristic peaks (Figure S5, Supporting Information). We also explored the release behaviors of Ce6 and PDE5‐i, and both release profiles exhibit approximately identical manners (Figure 2l,m). In detail, lower pH or NIR irradiation can drive Ce6 or PDE5‐i release, and the simultaneous presences of acidic microenvironment and NIR irradiation bring about the most Ce6 or PDE5‐i release (Figure 2l,m).

### 2.2 Photodynamic‐Derived ROS/RNS Production and Hypoxia Alleviation at the Cellular Level

The ubiquitous hypoxia in solid tumors still result in low ROS production efficiency in light of inadequate oxygen supply and the inherent resistances to ROS‐based therapeutic means.^[^ ^10^ ^]^ In an attempt to address these issues, great progress has been made to boost O_2_ release and augment ROS production for killing cancer cells.^[^ ^3^, ^9^ ^]^ Enlightened by this, the abundant O_2_ from this oxygen reservoir (i.e., Ce6/PDE5‐i@FHMON‐O_2_) is accessible and will pave a solid foundation to hypoxic microenvironment alleviation and intracellular ROS production. To assess it, hypoxic MCF‐7 cell model was established and used in all in vitro experiments. Herein, CoCl_2_ with varied concentrations were incubated with normal MCF‐7 cells to switch them into hypoxic ones,^[^ ^11^ ^]^ and the cells treated with 50 × 10^−6^ m CoCl_2_ were selected as the optimal model due to the highest hypoxia level (Figure S6, Supporting Information).

**Figure 3:**
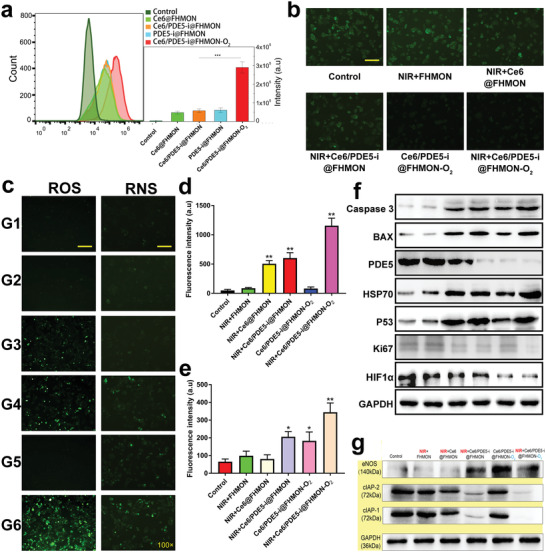
Principle validations of O_2_ release for mitigating hypoxia and enhancing ROS, and ROS conversion into RNS. a) Typical FCM patterns (right) and quantitative fluorescence intensities (left) of hypoxic MCF‐7 cells after incubating with different samples (dose: 200 µg mL^−1^ FHMON) labeled by FITC for evaluating their engulfment by hypoxic MCF‐7 cells. b) FIM images of hypoxic MCF‐7 cells that experienced different treatments after hypoxia probe (Hypoxyprobe Green) staining for tracking hypoxia alleviation, and scale bar = 50 µm. c) FIM images of hypoxic MCF‐7 cells that experienced different treatments after ROS probe (DCFH‐DA) and RNS probe (DAF‐AM DA) staining which are used monitor ROS and RNS levels, respectively, and scale bar = 100 µm. d,e) Quantitative fluorescence intensities of ROS and RNS in hypoxic MCF‐7 cells obtained according to image (c). f,g) Expression levels of different proteins in hypoxic MCF‐7 cells that experienced different treatments via WB analysis. Note, NIR parameter: 660 nm, 0.1 W cm^−2^, pulsed irradiation for 5 min in total with 5 cycles and 1 min interval between two cycles. Data are expressed as mean ± SD (*n* = 3). Statistical analyses were performed using a Student's *t*‐test in Prism software, and *P ˂ 0.05, **P ˂ 0.01, and ***P ˂ 0.001.

In vitro phagocytosis behavior of Ce6/PDE5‐i@FHMON‐O_2_ by hypoxic MCF‐7 cells was firstly evaluated through flow cytometry (FCM) because high accumulation can guarantee massive ROS/RNS production and excellent antitumor outcome. The accumulation levels of several counterparts (i.e., PDE5‐i@FHMON, Ce6@FHMON, and Ce6/PDE5‐i@FHMON) are approximately identical, suggesting that the single‐ or dual‐loading of Ce6 and PDE5‐i fail to hurt the ability of FHMON vehicles to enter MCF‐7 cells. Astonishingly, once binding with O_2_, more photocleaved O_2_‐released nanoplatforms (i.e., PDE5‐i/Ce6@FHMON‐O_2_) are allowed to enter MCF‐7 cells, as evidenced by the strongest fluorescence intensity (**Figure** **3a**). This intriguing phenomenon can be partially ascribed to that O_2_ bubbles can mitigate hypoxia, normalize hypoxic cells, and makes the nanoparticles‐based O_2_ reservoir camouflaged into oxygen‐supplied nutrient that are indispensible for sustaining normal metabolism activities,^[^ ^12^ ^]^ resulting in massive nanoparticles’ capture by MCF‐7 cells. As well, O_2_ bubbles‐enhanced cavitation can enhance the intratumoral permeability to permit free entry of Ce6/PDE5‐i@FHMON‐O_2_, which, along with fluorocarbon chains‐enhanced endosomal escape,^[^ ^3^, ^13^ ^]^ also contributed to the enhanced accumulation of Ce6/PDE5‐i@FHMON‐O_2_. Thanks to the high accumulation and adequate O_2_ release, this nanoplatform brings about the considerably elevated hypoxia alleviation and reverses the hypoxia microenvironment even without NIR irradiation, as shown in fluorescence inverted microscope (FIM) images (Figure 3b; Figure S7, Supporting Information).

RNS is preferable than ROS since it shares longer lifetime than ROS and can evade the hypoxia‐induced resistance to ROS‐based antitumor,^[^ ^4^, ^5^, ^6^ ^]^ which is beneficial for the overall antitumor activity. Given that RNS birth derives from ROS fusion with NO (Figure 1b), high ROS level is indispensible for massive RNS production. Regarding this, the sufficient oxygen supply and the accompanied hypoxia mitigation in this photocleaved O_2_‐supplied nanoplatform are available for giving birth to more ROS. Consistent with ESR results, NIR + Ce6/PDE5‐i@FHMON‐O_2_ group (G6) receives the highest ROS level that is two times larger than NIR + Ce6/PDE5‐i@FHMON (G4) (Figure 3c,d). Consequently, the most RNS production is easily accessible to the group of NIR + Ce6/PDE5‐i@FHMON‐O_2_ due to the most ROS production in comparison to any other groups (Figure 3c,e), suggesting that partial ROS were successfully converted into RNS. Notably, another precursor of RNS, i.e., NO, is independent of NIR irradiation, and merely associates with spontaneously intracellular blockading of PDE5 pathway using PDE5‐i.^[^ ^6^ ^]^ As a result, Ce6/PDE5‐i@FHMON‐O_2_ and NIR + Ce6/PDE5‐i@FHMON harvest the approximately identical RNS level due to the identical PDE5‐i content, but both are much inferior to NIR + Ce6/PDE5‐i@FHMON‐O_2_. The high expressions of ROS and RNS enabled by Ce6/PDE5‐i@FHMON‐O_2_ in the presence of NIR irradiation promise the excellent antitumor outcome. The signal decay profiles of ROS and RNS based on fluorescence semiquantitative analysis show that after transient NIR irradiation, ROS and RNS levels in Ce6/PDE5‐i@FHMON‐O_2_ rise rapidly. As the time elapses, the relative level of ROS decreases more rapidly than RNS. In particular, ROS is almost completely annihilated and no signal is observed at 60 min, while RNS remains detectable after 120 min (Figure S8, Supporting Information).

To uncover the underlying principles of ROS/RNS pathway activation, comprehensive western blot (WB) analysis was carried out. Results reveal that the introduction of PDE5‐i successfully blockade PDE5 target and cause PDE5 expression to drop in either PDE5‐i‐involved group (Figure 3f; Figure S9, Supporting Information), which denote that the cGMP singling pathway was activated to elevate eNOS expression that is responsible for NO production (Figure 3g; Figure S10, Supporting Information). Griess assay also confirms that the PDE5‐i‐involved groups (e.g., NIR + Ce6/PDE5‐i@FHMON, Ce6/PDE5‐i@FHMON‐O_2_, and NIR + Ce6/PDE5‐i@FHMON‐O_2_) harvest the considerably increased NO production in comparison to PDE5‐i‐free groups (e.g., Control, NIR + FHMON, NIR + Ce6@FHMON), as evidenced in Figure S11 of the Supporting Information. This appealing phenomenon explains why PDE5‐i‐involved groups evidently evoke massive RNS production especially for NIR + Ce6/PDE5‐i@FHMON‐O_2_. The variation trends of PDE5 inhibition and eNOS expression in different groups are approximately identical to that of ROS and RNS levels (Figure 3c–g; Figures S9 and S10, Supporting Information), i.e., Control ˂ NIR + CFHMON ˂ NIR + Ce6@FHMON ˂ NIR + Ce6/PDE5‐i@FHMON ˂ Ce6/PDE5‐i@FHMON‐O_2_ ˂ NIR + Ce6/PDE5‐i@FHMON‐O_2_. To deeply unravel hypoxia alleviation by O_2_ burst from Ce6/PDE5‐i@FHMON‐O_2_, HIF1*α* that is regarded as the typical hallmark of hypoxia was monitored.^[^ ^10a^ ^]^ It is found that although both O_2_‐carried groups give rise to the decreased HIF1*α* expression, NIR + Ce6/PDE5‐i@FHMON‐O_2_ outweighs Ce6/PDE5‐i@FHMON‐O_2_ in decrease HIF1*α* expression due to the more O_2_ release triggered by NIR (Figure 3f; Figure S9, Supporting Information).

### 2.3 In Vitro Antitumor Test via the Photodynamic‐Derived ROS/RNS Pathways Activation

**Figure 4:**
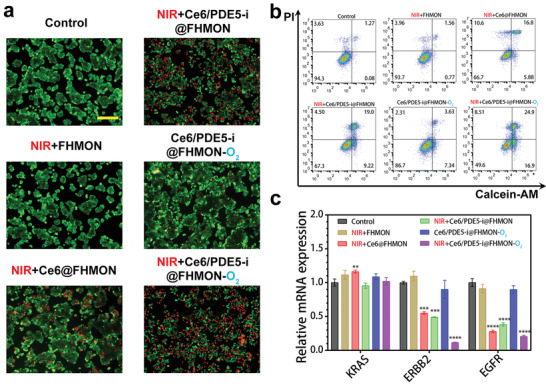
In vitro antitumor evaluations using this photocleaved O2‐released nanoplatform. a) FIM images and b) FCM patterns of hypoxic MCF‐7 cells that experienced different treatments after PI and calcein costaining and PI and Annexin V FITC costaining, respectively, and Sale bar = 200 µm. c) Relative mRNA expression levels of different genes (e.g., KRAS, ERBB2, and EGFR) via qRT‐PCR test in hypoxic MCF‐7 cells that experienced different treatments. Note, NIR parameter: 660 nm, 0.1 W cm^−2^, pulsed irradiation for 5 min in total with 5 cycles, and 1 min interval between two cycles. Data are expressed as mean ± SD (*n* = 3). Statistical analyses were performed using a Student's *t*‐test in Prism software, and **P˂ 0.01, ***P ˂ 0.001, and ****P ˂ 0.0001. Dose: 200 µg mL^−1^ FHMON.

Subsequently, in vitro antitumor explorations were implemented since the two concerns, which are low ROS production efficiency and SLT, were successfully addressed by O_2_ burst and ROS switching into RNS, respectively. To identify dead and live cells, hypoxic MCF‐7 cells were costained by PI and calcein AM after experiencing different treatments and subsequently traced by FIM (**Figure** **4a**). Both Ce6/PDE5‐i@FHMON‐O_2_ and Ce6/PDE5‐i@FHMON in the presence of NIR result in a large number of cell apoptosis in comparison to other groups. In particular, the largest magnitude of hypoxia alleviation and the most productions of ROS and RNS enable NIR + Ce6/PDE5‐i@FHMON‐O_2_ to acquire the most apoptotic cells. Identical results were obtained via FCM analysis wherein different death stages were meticulously identified by costaining of propidium iodide (PI) and annexin V‐ FITC (Figure 4b; Figure S12, Supporting Information). Encouraged by the appealing result that hypoxic MCF‐7 cells actively engulfed massive Ce6/PDE5‐i@FHMON‐O_2_, the largest apoptosis including early and late parts occur to NIR + Ce6/PDE5‐i@FHMON‐O_2_. More significantly, RNS pathway activation along with ROS elevation, hypoxia mitigation, resistance liberation, contributed to the most considerably enhanced antitumor consequences. By virtue of these distinctive characters of Ce6/PDE5‐i@FHMON‐O_2_ in the presence of 660 nm laser irradiation, CCK8 test also reflects the best antitumor outcome in vitro (Figure S13, Supporting Information).

To figure out the apoptosis principle, some common proapoptotic and antiapoptotic proteins were examined via WB analysis. The expressions of some proapoptotic proteins including BAX, Caspase‐3, HSP70, and P53 are progressively upregulated and the validation trend follows that of cell apoptosis in FIM and FCM (Figure 3f; Figure S9, Supporting Information), indicating these apoptotic pathways were activated. Accordingly, antiapoptotic proteins such as cIAP‐1 and cIAP‐2 accordingly recede and NIR + Ce6/PDE5‐i@FHMON‐O_2_ receives the lowest expression of cIAP‐1 and cIAP‐2 (Figure 3g; Figure S10, Supporting Information), concurrently accompanied by the strongest cell proliferation inhibition associated with the lowest Ki67 expression (Figure 3f; Figure S9, Supporting Information). To comprehensively understand the apoptosis, deep mechanistic investigation via supervising gene expression was carried out using polymerase chain reaction (PCR) technology. The NIR + Ce6/PDE5‐i@FHMON‐O_2_ treatment alters the mutation of cancer genes (ERBB2 and EGFR) and decreases their expressions (Figure 4c), which reverses genetic code of tumor growth at source.

### 2.4 In Vivo Antitumor Evaluations Using Photodynamic‐Derived ROS/RNS‐Based Strategy in Ce6/PDE5‐i@FHMON‐O_2_

The unprecedented conversion from ROS to RNS can address the SLT of ROS via activating RNS pathway, which bypass rapid ROS annihilation. Inspired by the outstanding antitumor outcomes in vitro, in vivo antitumor can also be expected. Prior to in vivo assessment, the biocompatibility of this photocleaved O_2_‐released nanoplatform was explored. In short, in vitro hemolysis test and cytotoxicity test show the safety of this nanoplatform (Figures S14 and S15, Supporting Information). The routine blood assay manifests neglectable variations of blood and biochemical indexes in comparison to control (Figure S16, Supporting Information). After 30 days postinjection of this nanoplatform, no evident injures to normal organs further validate the in vivo biosafety of this nanoplatform via H&E microscopic observation (Figure S17, Supporting Information).

**Figure 5:**
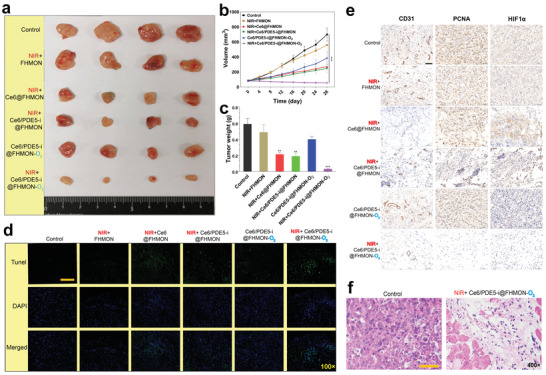
In vivo antitumor experiments and mechanistic explorations using this nanoplatform on MCF‐7 breast cancer‐bearing nude mice. a) Digital photos of harvested MCF‐7 breast tumors in each group at the end of experiment period (Day 28). b) Time‐dependent variation profiles of MCF‐7 tumor volume implanted on nude mice that experienced different treatments, e.g., Control, NIR + FHMON, NIR + Ce6@FHMON, NIR + Ce6/PDE5‐i@FHMON, Ce6/PDE5‐i@FHMON‐O_2_, and NIR + Ce6/PDE5‐i@FHMON‐O_2_. c) The weights of MCF‐7 tumors harvested from MCF‐7 breast cancer‐bearing nude mice that experienced aforementioned different treatments at the end of experiment period (Day 28). d) TUNEL immunofluorescence images and e) CD31, PCNA, and HIF1*α* immunohistochemical images of tumor slices harvested from MCF‐7 breast cancer‐bearing nude mice that experienced aforementioned different treatments at the end of experiment period (Day 28), scale bar = 50 µm. f) H&E optical microscopic images of tumor slices in two groups, i.e., Control and NIR + Ce6/PDE5‐i@FHMON‐O_2_, scale bar = 50 µm. Note, NIR parameter: 660 nm, 0.65 W cm^−2^, pulsed irradiation for 30 min per day in total with 6 cycles, and 5 min interval between two cycles, and repeated irradiations were enforced per three days. Data are expressed as mean ± SD (*n* = 4). Statistical analyses were performed using a Student's *t*‐test in Prism software, and **P ˂ 0.01 and ***P ˂ 0.001. Dose: 100 mg FHMON/KG mice.

Before evaluating the animal treatment model in vivo, we first evaluated the retention effect of Ce6/PDE5‐i@FHMON‐O_2_ in tumor by animal fluorescence imaging. Ce6/PDE5‐i@FHMON‐O_2_ nanparticles are gradually enriched in tumor based on classic enhanced permeability and retention effect. Once combining with NIR irrasiation, NIR‐induced mild heat and O_2_ burst‐enhanced cavitation can further enhance the vascular permeability and allow more nanoparticles to enter and retain in tumor (Figure S18a,b, Supporting Information). In addition, fluorescence images of ex vivo tumor and other normal organs also indicate that NIR irradiation promotes more accumulations of Ce6/PDE5‐i@FHMON‐O_2_ nanparticles in tumor in comparison to Ce6/PDE5‐i@FHMON‐O_2_ alone (Figure S18c, Supporting Information). In in vivo antitumor experiments, either NIR + Ce6@FHMON or NIR + Ce6/PDE5‐i@FHMON treatment can delay tumor growth to some extent. Nevertheless, both groups fail to shrink the tumor due to the low levels of ROS alone and ROS/RNS, respectively. The tumor volume slowly increases as the incubation period is prolonged (**Figure** **5a**,b). By contrast, only NIR + Ce6/PDE5‐i@FHMON‐O_2_ treatment shrinks tumors and brings about the most robust inhibitory effect against hypoxic MCF‐7 breast cancer with the smallest weight (Figure 5a–c), wherein the treated tumors gradually recede as the time rises (Figure 5b). No evident difference in weight during treatment process is observed (Figure S19, Supporting Information).

As well, systematic pathological examinations were enforced to comprehend the treatment mechanism after different immunohistochemical and immunofluorescence staining of treated tumors that were isolated and sliced. The most apoptotic cells were observed in NIR + Ce6/PDE5‐i@FHMON‐O_2_ after TUNEL immunofluorescence staining (Figure 5d; Figure S20, Supporting Information). Simultaneously, the NIR + Ce6/PDE5‐i@FHMON‐O_2_ treatment represses angiogenesis and tumor cell proliferation, as evidenced by the lowest expressions of CD31 and proliferating cell nuclear antigen (PCNA) (Figure 5e), respectively. As well, some apoptosis characteristics such chromatin pyknosis, nuclei rupture, and cell density decrease also uncover the in vivo antitumor mechanism using this photocleaved O_2_‐released nanoplatform in the presence of NIR irradiation (Figure 5f). Intriguingly, O_2_‐contained groups can rapidly mitigate hypoxia in MCF‐7 solid tumors (Figure S21, Supporting Information). Especially when NIR irradiation is added, the treatment in NIR + Ce6/PDE5‐i@FHMON‐O_2_) group brings about the lowest hypoxia level. From the pathological examination, the photocleaved O_2_ burst from Ce6/PDE5‐i@FHMON‐O_2_ significantly decrease HIF1*α* expression (Figure 5e), consequently reversing the hypoxic zone in MCF‐7 solid tumor. In short, the antitumor using NIR + Ce6/PDE5‐i@FHMON‐O_2_ treatment follow the pathway that is hypoxia mitigation, cell apoptosis, cell proliferation termination, revascularization disruption, and cell density decrease. The largest degree of ROS/RNS pathway activations due to the most productions of ROS and RNS in NIR + Ce6/PDE5‐i@FHMON‐O_2_ is responsible for these striking results. As well, an appealing phenomenon that FHMON carriers are degraded at 4 °C after 26 days is observed (Figure S22, Supporting Information), which indicates that more rapid biodegradation in living body can be expected to potentiate its clinical translation.

## 3 Conclusion

In summary, we established a novel photocleaved O_2_‐released nanoplatform to activate ROS/RNS pathways and address the low yield efficiency and SLT of ROS via providing sufficient O_2_ and converting partial ROS into RNS featuring longer lifetime than ROS. Thanks to the adequate photocleaved O_2_ supply, this nanoplatform was demonstrated to alleviate hypoxic microenvironment and yield abundant ROS for inhibiting the hypoxic breast tumor in vitro and in vivo. More significantly, the entrapped PDE5‐i could inhibit PDE5 pathway to upregulate eNOS for activating massive NO production, which allowed ROS to combine with NO and transformed ROS into RNS for further contributing to the considerably repressed tumor growth. The activated RNS pathway via transforming ROS into RNS provides a general method to address the fatal drawback of ROS (i.e., SLT). In particular, the underlying principles that involve O_2_ production and activated ROS/RNS pathways will also act as a general guidance approach for designing other ROS‐based nanoplatforms, e.g., SDT‐based nanoplatforms, CDT‐based nanoplatforms.

## 4 Experimental Section

All experimental details, methods, parameters, and supplementary figures were provided in the Supporting Information.

## Conflict of Interest

The authors declare no conflict of interest.

## Supporting information

Supporting Information

## Data Availability

The data that support the findings of this study are available on request from the corresponding author.
