# Supplementary material for: Switching Reactive Oxygen Species into Reactive Nitrogen Species by Photocleaved O2‐Released Nanoplatforms Favors Hypoxic Tumor Repression
Source: Adv Sci (Weinh). 2021 Aug 8;8(19):2101065. doi: 10.1002/advs.202101065 (PMC8498884; doi:10.1002/advs.202101065)
Supplement: Supplementary file 1 — Supporting Information [file ADVS-8-2101065-s001.pdf]

© 2021 Wiley-VCH GmbH

ADVANCED SCIENCE
Open Access

Supporting Information

for *Adv. Sci.*, DOI: 10.1002/advs.202101065

Switching Reactive Oxygen Species into Reactive Nitrogen Species by Photo-Cleaved O_2-Released Nanoplatforms Favors Hypoxic Tumor Repression

Tao Luo, Duo Wang, Lidong Liu, Yan Zhang, Chuangye Han, Ying Xie, Yan Liu, Jingchen Liang, Guanhua Qiu, Hongxue Li, Danke Su, Junjie Liu* and Kun Zhang**

Supporting Information for

Switching Reactive Oxygen Species into Reactive Nitrogen Species by Photo-Cleaved O_2-Released Nanoplatforms Favors Hypoxic Tumor Repression

Tao Luo,[#] Duo Wang,[#] Lidong Liu,[#] Yan Zhang, Chuangye Han, Ying Xie, Yan Liu, Jingchen Liang, Guanhua Qiu, Hongxue Li, Danke Su, Junjie Liu* and Kun Zhang**

Methods

Synthesis of Ce6/PDE5-i@FHMON-O_2 and its immediates (*e.g.*, FHMON, Ce6@FHMON, PDE5-i@FHMON, Ce6/PDE5-i@FHMON)

FHMON carriers were obtained according to a previously-reported classic method. In detail, absolute ethyl alcohol (36 mL), deionized water (5 mL) and ammonia solution (1.6 mL) were added and completely mixed in the round-bottom flask (250 volume) *via* magnetic stirring (velocity: 430 rpm) at 30 °C for 20 min. Afterwards, tetraethyl orthosilicate (3 mL) was dropwise injected during magnetic stirring (velocity: 430 rpm), and reacted for 45 min. Following that, a completely-mixed solution containing TEOS (2.5 mL), PDES (1 mL) and ethyl alcohol (0.5 mL) was injected into above dispersion at the injection velocity of 100 μL/min, and continued to react for another 40 min. Subsequently, aforementioned dispersion was averaged into two centrifuge tubes and washed with deionized water for several times for use. Each one was re-dispersed in Na_2CO_3 aqueous solution (75 mL, 0.6 M) and placed in water bath for 6 min at 60 °C. Eventually, The FHMON carriers were collected *via* twice different centrifugations in sequence, *i.e.*, 3000 rpm for 5 min and 5000 rpm for 5 min.

As for preparing Ce6@FHMON, PDE5-i@FHMON and Ce6/PDE5-i@FHMON, 100 mg FHMON carriers were dispersed in 20 mL of DMSO containing Ce6 or/and PDE5-i (10 mg/mL) and

stirred for overnight at room temperature. After that, the samples were collected and washed with water three times *via* three repeated centrifugations (1200 rpm, 8 min), and then dispersed in PBS and stored at 4 °C for use. As for Ce6/PDE5-i@FHMON-O_2 synthesis, O_2 bubbling for 2 h were implemented in Ce6/PDE5-i@FHMON solution with varied concentrations according to detailed experiment demands, and O_2-adsorbed nanoplatforms were collected *via* rapid centrifugation (1200 rpm, 5 min) for immediate use.

Material characterizations

Transmission electron microscope (TEM) images and scanning electron microscopy (SEM) images were obtained on -FEI Tecnai F20 (FEI, America) and SU8020 (HITACHI, Japan), respectively. UV-Vis spectra and FTIR spectra were recorded on UV-3600 (Shimadzu, Japan) and Nicolet IS10, (Thermo Nicolet Corporation, America), respectively; N_2 adsorption and desorption isotherms and pore diameter distribution was measured on ASAP 2460 (Micromeritics America), dynamic light scattering (DLS) and zeta potential determination were carried on Zetasizer Nano S (Malvern, UK), ultrasound imaging and electron spin resonance (ESR) spectra were obtained on , LOGIQ E9 (GE company) and EMXplus EMXmicro (Bruker, Germany), respectively. XPS spectra were recorded on Thermo Scientific™ K-Alpha™ (Thermo Fisher Scientific, America).

Loading and release of O_2 from Ce6/PDE5-i@FHMON-O_2

The O_2 loading amount in Ce6/PDE5-i@FHMON-O_2 was determined by Gradient dissolved oxygen method. In detail, 50 mg of Ce6/PDE5-i@FHMON-O_2 were dispersed in deionized water with varied volumes (*e.g.,* 0.1 L, 0.5 L, 1.0 L, 2.0 L, 3.0 L, 5.0 L and 7.0 L) and sealed. These water-contained bottles were heated at 50 °C for 8 h, respectively. Afterwards, unisense oxygen microelectrode was used to detect the dissolved O_2 concentration and calculated the released O_2 content. Once the O_2

content reached a saturated plateau, the corresponding O_2 content was regarded as the loading amount of Ce6/PDE5-i@FHMON-O_2 (50 mg).

As well, O_2 release *in vitro* was evaluated. Six groups (*i.e.*, Control, NIR+FHMON, NIR+Ce6@FHMON, NIR+Ce6/PDE5-i@FHMON, Ce6/PDE5-i@FHMON-O_2 and NIR+Ce6/PDE5-i@FHMON-O_2) were classified, wherein the concentration of FHMON in water was set as 10 mg/mL in all groups. Dissolved oxygen concentration was detected using unisense oxygen microelectrode at 0 h, 0.5 h, 1 h, 2 h, 3 h, 5 h, 8 h and 10 h, respectively.

Release of Ce6 and PDE5-i from Ce6/PDE5-i@FHMON-O_2

Phosphate buffer solution (PBS) with varied pH values at 7.4 and 6.0 were set, and three circumstances (*i.e.*, PBS (pH=7.4), PBS (pH=6.0), NIR + PBS (pH=6.0)) were divided. 5 mg of Ce6/PDE5-i@FHMON-O_2 was added to dialysis bags, and then these dialysis bags were placed into sealed centrifuge tube containing 25 mL of above different media corresponding to different circumstances. Afterwards, the sealed centrifuge tubes were vibrated in table concentrator at an oscillation rate of 200 rpm. In the NIR + PBS (pH=6.0), NIR radiation was carried out at four time points (*i.e.*, 0 h, 1 h, 3 h, 6 h) within 12 hours. During the experiment, the intensity of characteristic peaks of Ce6 and PDE5-i were monitored on UV-3600 (Shimadzu, Japan) at certain intervals (*e.g.*, 0 h, 0.5 h, 1 h, 2 h, 3 h, 5 h, 8 h, 10 h, 12 h), and the release curves of Ce6 and PDE5-i could be obtained according to their concentration-absorbance standard curves, respectively.

Cell lines and animals

Human breast cancer cell lines MCF-7, human umbilical vein endothelial cells (HUVECs), and human diploid fibroblasts (HDFs) were purchased from the ATCC (Manassas, VA, USA) and cultured in Dulbecco's modified Eagle's medium (DMEM) (HyClone, Logan, UT, USA)

supplemented with 10% fetal bovine serum (FBS) (HyClone, Logan, UT, USA) and 100 U/mL penicillin–streptomycin (HyClone, Logan, UT, USA). The cells were maintained in a humidified 37 °C incubator with a 5% CO_2 atmosphere. Nude mice (4 weeks) were maintained under the protocols approved by affiliated Cancer Hospital of Guangxi Medical University Laboratory Animal Center with an approval number (LW2019062).

Hypoxic MCF-7 tumor model establishment

MCF-7 cells (1) were incubated with $CoCl_2$-contained culture media with varied $CoCl_2$ concentrations (0, 30, 50 and 100 μM), and after 12 h incubation, the cells were collected and stained by anti-HIF1α antibody. Western blot images were analyzed semi-quantitatively using Quantity One software (Bio-Rad Laboratories). The relative intensity values of bands were normalized to GAPDH.

RNA isolation, reverse transcription and quantitative real-time polymerase chain reaction (qRT-PCR)

Total RNAs were extracted from cell lines using Trizol reagent (Thermo Scientific, USA) according to the manufacturer's procedures. Total RNA (500 ng) was reverse transcribed using SuperScript II Reverse Transcriptase to obtain complementary DNA. PCR was carried out with SYBR green PCR Master Mix following standard protocols. Quantitative real-time PCR analysis was done on a MyiQ real-time PCR cycler (BioRad). β-Actin was used as the internal control.

Western blot analysis

MCF-7 were inoculated with 6-well plates according to 2×10^5 cells/well and incubated in CO_2 incubator for 24 h. The $CoCl_2$ solutions of 30 μM, 50 μM and 100 μM were added to the 6-well plates and blank wells were set up. After another 24 h, the samples were collected. For protein detection, the cells were lysed by ice-cold RIPA buffer (Beyotime Biotechnology, Shanghai, China)

and centrifuged at 4 °C with a speed rate of 12,000 ×g for 5 min. Protein concentrations were determined by BCA (Beyotime Biotechnology, Shanghai, China). Total proteins (20 μg/lane) were subjected to electrophoresis in 12% polyacrylamide gel, followed by transferring to a polyvinylidene difluoride membrane. Immediately afterwards, the membrane was blocked at 37 °C for 1 h with 5% non-fat milk in tris-buffered saline containing 0.05% Tween-20. The blots were then separately incubated with primary antibodies for HIF1α (1:1000; ab51608; Abcam, USA), HRP-Conjugated GAPDH Antibody (1:5000; HRP-60004; proteintech, USA), Anti-Mouse IgG, HRP-linked Antibody (1:1000; 7076P2; Cell Signaling Technology, USA), Signals were detected by enhanced chemiluminescence (Thermo Fisher Scientific). Western blot images were analyzed semi-quantitatively using Quantity One software (Bio-Rad Laboratories). The relative intensity values of bands were normalized to GAPDH.

Intracellular endocytosis by FCM

To detect the phagocytosis, the cells were treated according to the following groups (Control, Ce6@HMON-FITC, Ce6/PDE5-i@HMON-FITC, PDE5-i@HMON-FITC, Ce6/PDE5-i@HMON-O_2-FITC). 10^5 cells in each group were centrifuged at 1200 rpm for 5 min, and the supernatant was removed. 200 μL of PBS was added respectively, and the phagocytosis rate of cells was detected by flow cytometry (BD Accuri™ C6 PLUS, China) within 0.5 h.

FCM analysis for determining early and late apoptosis

To detect the apoptotic rate, six groups (*i.e.*, Control, NIR+FHMON, NIR+Ce6@FHMON, NIR+Ce6/PDE5-i@FHMON, Ce6/PDE5-i@FHMON-O_2, NIR+Ce6/PDE5-i@FHMON-O_2) were prepared. After washing with PBS, fluorescein isothiocyanate (FITC)-labeled Annexin V (5 μL) and PI (5 μL) was added into the cells and incubated in the dark for 15 min at 37 °C. The cell apoptosis

rate was analyzed by flow cytometry within 1 h.

CCK8 assay for evaluating viability

Cell proliferation was evaluated with a Cell Counting Kit-8 (CCK8, Tongren, China) as the manufacturer's solution. Six groups (*i.e.*, Control, NIR+FHMON, NIR+Ce6@FHMON, NIR+Ce6/PDE5-i@FHMON, Ce6/PDE5-i@FHMON-O_2, NIR+Ce6/PDE5-i@FHMON-O_2) were prepared. Then CCK8 solution (10 μL) was added into each pore, incubated at 37 °C for 100 min, and then measured the absorbance at 450 nm on thermo enzyme reader (Thermo Scientific, USA).

Cell hypoxia alleviation detection

Hypoxyprobe™ Green Kit (HP6, Hypoxyprobe, INC) was used to measure the hypoxia in each group of the hypoxic MCF-7 cell model. Six groups (*i.e.*, Control, NIR+FHMON, NIR+Ce6@FHMON, NIR+Ce6/PDE5-i@FHMON, Ce6/PDE5-i@FHMON-O_2, NIR+Ce6/ PDE5-i@FHMON-O_2) were set to detect *in vitro* hypoxia. In brief, $CoCl_2$ (50 μM) and hypoxia probe hypoxyprobe-1 200 μM were added to the cell culture medium of each group and incubated for overnight, and another pulsed irradiation was enforced under 660 nm laser with power density of 0.1 W/cm^2 with 5 cycles, 1 min interval between two cycles and 5 min in total. 4% paraformaldehyde was added to fix them for 30 minutes, then sealed it with 1% BSA for 1 h, and incubated with 100x diluted FITC-MAb1 at room temperature for overnight. The cells were stained with DAPI for 3 min and then washed with PBS (pH=7.4) three times. The cytoplasmic green fluorescence was observed at the excitation wavelength of 488 nm and emission wavelength of 530 nm.

Double staining of calcein AM/PI cells for differentiating dead and live cells

The survival/necrosis rate of cells in each group were measured after drug treatment (dose: 200

μg/mL FHMON) and laser irradiation. The group setting was the same as above, $CoCl_2$ (50 μM) and hypoxia probe (*i.e.*, hypoxyprobe-1, 200 μM) were added to the cell culture medium of each group, incubated overnight, and afterwards NIR laser irradiation was carried out, where NIR parameters: 660 nm, 0.1 W/cm^2, pulsed irradiation for 5 min in total with `5 cycles and 1 min interval between two cycles. The PBS buffer containing Calcein-AM (C542, Dojindo, Shanghai, China) (0.8 μM) and PI (C542, Dojindo, Shanghai, China) (1.5 μM) was added to the cells and incubated in a 5% CO_2 incubator for 15 min at 37 °C three times. The cytoplasmic green fluorescence was observed at the excitation wavelength of 490 nm and emission wavelength of 515 nm, and red fluorescence was observed at the excitation wavelength of 530 nm and emission wavelength of 580 nm.

***In vitro* NO detection**

Griess reagent kit was used to determine NO production. In detail, the hypoxic MCF-7 cells were seeded in a 6-well plate. Six groups (*i.e.*, Control, NIR+FHMON, NIR+Ce6@FHMON, NIR+Ce6/PDE5-i@FHMON, Ce6/PDE5-i@FHMON-O_2 and NIR+Ce6/PDE5-i@FHMON-O_2) were set, and the corresponding treatment in different groups were carried out. Herein, NIR laser parameters were fixed as follows: 660 nm, 0.1 W/cm^2, pulsed irradiation for 5 min in total with `5 cycles and 1 min interval between two cycles. After incubation for 30 min, the cells were lysed and the cell supernatant was extracted by centrifugal precipitation. After that, 50 μL of Griess Reagent I and Griess Reagent II were added into each well. Then the absorbance at 540 nm was recorded on thermo enzyme reader (Thermo Scientific, USA).

***In vitro* RNS test *via* fluorescence inverted microscope (FIM)**

To uncover the underlying mechanism of how NIR+Ce6/PDE5-i@FHMON-O_2 nanomaterials killed tumor cells, the intracellular RNS production level was detected by BBoxiProbe™ R21

(BBoxiProbe™, BB470507, BestBio, Shanghai, China), which is the latest generation of fluorescent probe for quantitative RNS detection *in vitro*. The specific experimental group and pre-cell treatment are the same as above, where NIR parameters: 660 nm, 0.1 W/cm^2, pulsed irradiation for 5 min in total with `5 cycles and 1 min interval between two cycles. Cell culture medium was removed and diluted BBoxiProbe™ (5 μmol/L) was added. The added volume was adjusted to completely cover the cells, and the volume of diluted BBoxiProbe™ that was added to each well in the six-well plate was usually 1 mL. The cells were incubated in a cell incubator at 37 °C for 20 min. Washing the cells with PBS (pH = 7.4) three times was carried out to completely remove the residual BBoxiProbe™ that failed to enter the cells. Afterwards, the cytoplasmic green fluorescence was observed on the fluorescence inverted microscope (FIM, Sedorius, E200, Germany) with an excitation wavelength at 495 nm and emission wavelength at 515 nm.

***In vitro* ROS detection *via* FIM**

Reactive oxygen species detection kit was used to determine the expression of *intracellular* reactive oxygen species after different treatments (dose: 200 μg/mL FHMON), wherein NIR laser parameters: 660 nm, 0.1 W/cm^2, pulsed irradiation for 5 min in total with `5 cycles and 1 min interval between two cycles. The specific experimental groups and pre-cell treatment were the same as above. After 660 nm NIR irradiation, the cell culture medium was removed, and the medium containing 1/1000 diluted reactive oxygen species detection reagent DCFH-DA (10 μM, S0033, Beyotime, Shanghai, China) was added and incubated at 37 °C in 5% CO_2 incubator for 15 minutes, followed by washing with PBS three times. Then the cytoplasmic green fluorescence was observed on the FIM (Sedorius, E200, Germany) with an excitation wavelength at 488 nm and emission wavelength at 525 nm.

Relative semi-quantitative evaluations on the lives of ROS and RNS

Hypoxic MCF-7 cells were seeded in 96-well plates (5000 cells per well). After 24 h, ROS indicator (DCFH-DA, S0033, Beyotime, Shanghai,China）and RNS indicator (BBoxiProbeTM, BB470507, BestBio, Shanghai, China) were added, respectively, and incubated with cells for 20 min. Immediately afterwards, the excessive dyes were sucked out, and Ce6/PDE5-i@FHMON-O_2 dispersion was added, accompanied which NIR irradiation (660 nm, 0.1 W/cm^2, 1 min) was carried out. Eventually, microplate reader (Varioskan LUX,ThermoFisher scientific,China) was used to detect the fluorescence absorbance.

Toxicity test of FHMON *in vitro*

HUVECs and HDFs cells were seeded in 96-well plates (5000 cells per well). After incubation for 24 h, FHMON nanoparticles were added according to the concentrations of 0, 25, 50, 100, 200, 300, and 500 μg/mL. After another incubation for 24 h, the supernatant was extracted and discarded, and then CCK8 solution (10 μL) was added into each well. After incubation at 37 °C for 100 min, the absorbance was measured at 450 nm on thermo enzyme reader (thermo scientific, USA), and the optical density (OD) value was recorded. Cell survival rate = $(OD_{\text{the experiment group}} - OD_{\text{the blank group}})/(OD_{\text{the control group}} - OD_{\text{the blank group}}) \times 100\%$.

Evaluations on blood compatibility *via* routine blood analysis

The whole blood of adult SD rats was added with 5 mL of lymphocyte separation solution and 500 μL of sample diluents to keep the liquid clear. Subsequently, the mixture was centrifuged (800 g) at room temperature for 30 min, and the blood cell layer was slowly suck out with a liquid transfer gun and then washed with 10 mL medium; followed by further centrifugation (250 g) for 10 min at room temperature. The cells were collected, and resuscitated with distilled water, PBS, normal saline and nanoparticles for 1 h. Smear after centrifugation, filter paper sucked dry and sealed, then detected by

biological fluorescence inverted microscope (Sedorius, E200, Germany).

In vivo animal fluorescence imaging on MCF-7 hypoxic tumor

The animal experiments were approved by the Institutional Animal Care and Use Committee of affiliated Cancer Hospital of Guangxi Medical University in accordance with the Guide for the Care and Use of Laboratory Animals with an approval number (LW2019062). Four-week-old athymic nude mice were supplied by experimental animal center of affiliated Cancer Hospital of Guangxi Medical University. All the tumor-bearing mice were randomly divided into two groups (n = 3) for different treatments: NIR+Ce6@FHMON-O_2 and Ce6@FHMON-O_2, where pulsed 660 nm laser irradiation (0.65 W/cm^2) was carried out for 15 minutes in total with 3 cycles and 2 minutes interval between two cycles. At 0.5 h, 1 h, 2 h, 4 h and 6 h, respectively, fluorescence images were captured. After 6 hours post-treatment, nude mice were sacrificed and some main organs were collected to observe the biological distribution of nanoparticles in tumor, heart, liver, spleen, lung and kidney.

Biosafety evaluations

The animal experiments were approved by the Institutional Animal Care and Use Committee of affiliated Cancer Hospital of Guangxi Medical University in accordance with the Guide for the Care and Use of Laboratory Animals with an approval number (LW2019062). MCF-7 tumor-bearing nude mice were supplied by experimental animal center of affiliated Cancer Hospital of Guangxi Medical University. Mice were divided into two groups (n = 5), *i.e.*, control and NIR+Ce6/PDE5-i@FHMON-O_2, wherein NIR represents pulsed 660 nm laser irradiation (0.65 W/cm^2) for 30 minutes per day in total with 6 cycles and 5 minutes interval between two cycles, and repeated irradiations were enforced per two days (three irradiations in total). After 30 days, routine blood indexes were tested.

In vivo anti-tumor experiments

The animal experiments were approved by the Institutional Animal Care and Use Committee of affiliated Cancer Hospital of Guangxi Medical University in accordance with the Guide for the Care and Use of Laboratory Animals with an approval number (LW2019062). Four-week-old athymic nude mice were supplied by experimental animal center of affiliated Cancer Hospital of Guangxi Medical University. All the tumor-bearing mice were randomly divided into six groups (n = 4) for different treatments: (1) control, (2) NIR+FHMON, (3) NIR+Ce6@FHMONc, (4) NIR+Ce6/PDE5-i@FHMON, (5) Ce6/PDE5-i@FHMON-O_2, (6) NIR+Ce6/PDE5-i@FHMON-O_2. MCF-7 cells (5×10^6) were suspended in 50 μL of PBS and subcutaneously implanted in nude mice. When the tumor reached 70-100 mm^3 in diameter, the drug (dose: 100 mg FHMON/kg mice) was administered through the tail vein, and next day, pulsed 660 nm laser irradiation (0.65 W/cm^2) was carried out for 30 minutes per day in total with 6 cycles and 5 minutes interval between two cycles, and repeated irradiations were enforced per two days (three irradiations in total). The size of the xenograft tumor was measured every 4 days using a Vernier calliper, and the volume was calculated at different indicated intervals post-transplantation as follows: Volume = $W^2 \times L/2$, where W and L represent the width and length of tumor. The tumor was collected after 28 days, and the subcutaneous xenografts were weighed and analyzed by IHC staining, TUNEL staining and H&E staining.

Non-invasive detection of SpO2 in subcutaneously-transplanted tumors

The 4-week-old nude mice were subcutaneously injected with hypoxic MCF-7 cells. After the tumors grew to about 200 mm^3, MCF-7 bearing mice were randomly divided into 6 groups (n = 3), *i.e.*, Control, NIR+FHMON, NIR+Ce6@FHMON, NIR+Ce6/PDE5-i@FHMON, Ce6/PDE5-i@FHMON-O_2 and NIR+Ce6/PDE5-i@FHMON-O_2. At 0 h, 6 h, and 12 h, respectively,

the saturation of pulse oximetry (SpO_2) in tumor was detected on the clinical pulse oximeter (Beneview T1, Mindray).

CD31, PCNA and HIF1α immunohistochemical staining

Tissue sections were deparaffinized and hydrated using a xylene and series of graded alcohol treatments. The sections were incubated with rabbit polyclonal anti-HIF1α (1 : 100, ab51608, Abcam, Cambridge, UK), rabbit polyclonal anti-CD31 (1:2000, GB13428, Servicebio, Wuhan, China) and rabbit polyclonal anti-PCNA (1:500, GB11010, Servicebio, Wuhan, China) antibodies at 4 °C overnight, followed by subsequent incubation for 50min with goat anti-rabbit IgG-horseradish peroxidase for detection. DAB was used as a chromogen, the staining time was controlled under common optical microscope, and the positive was brownish yellow. Hematoxylin was re-stained on nucleus and then dehydrated and sealed.

TUNEL immunofluorescence staining

Dewaxing in xylene for 5-10 minutes was implemented and then switched to fresh xylene and dewaxed for 5-10 minutes, followed by the subsequent procedures in sequence: anhydrous ethanol for 5 minutes, 90% ethanol for 2 minutes, 70% ethanol for 2 minutes and distilled water for 2 minutes. Then operate according to the instructions of TUNEL apoptosis detection kit (C1089, Beyotime Biotechnology, Shanghai, China). The tablets were sealed with anti-fluorescence quenching solution and observed under IFM (Sedorius, E200, Germany). The excitation wavelength of Cy3 is 550 nm and the emission wavelength is 570 nm (red fluorescence).

H&E immunohistochemical staining

The paraffin slices were sequentially put into xylene I 20 min, xylene II 20 min, anhydrous ethanol I 5 min, anhydrous ethanol II 5 min, 75% alcohol 5 min, and eventually washed with tap water. Then

hematoxylin and eosin staining were performed respectively, and the tablets were dehydrated and sealed. Finally, it is examined by optical microscope, and the image is collected and analyzed.

Statistical analysis

Normalization was used to determine the cell viability and time-dependent ROS and RNS lifetimes. The results of quantitative experiments were presented as the mean value ± standard deviation (for all data, $n \geq 3$). The Student's two-tailed *t*-test was performed to compare two groups using GraphPad prism 8.0 software (GraphPad Inc., San Diego, CA, USA). Single, double, and triple asterisks represented $p < 0.05$, 0.01, and 0.001, respectively, and $p < 0.05$ was set as the significance threshold. NS, not significant.

Supplementary figures

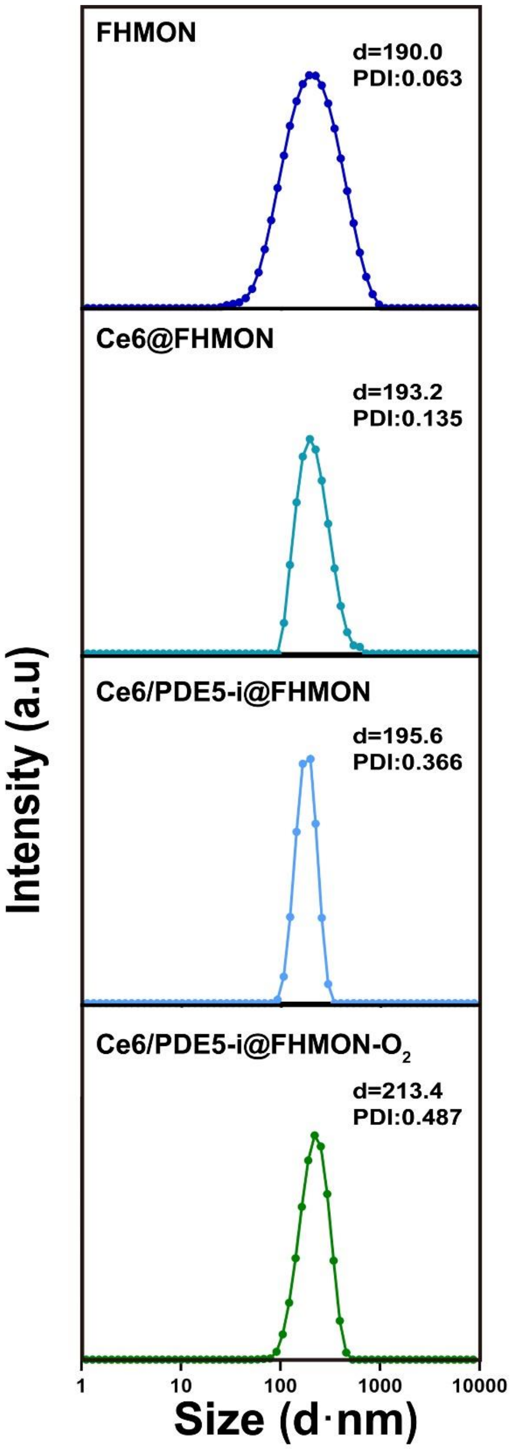

Figure S1 Particle size distributions of different samples, *e.g.*, FHMON, Ce6&FHMON, Ce6/PDE5-i@FHMON, and Ce6/PDE5-i@FHMON-O_2, which are determined by dynamic light scattering (DLS).

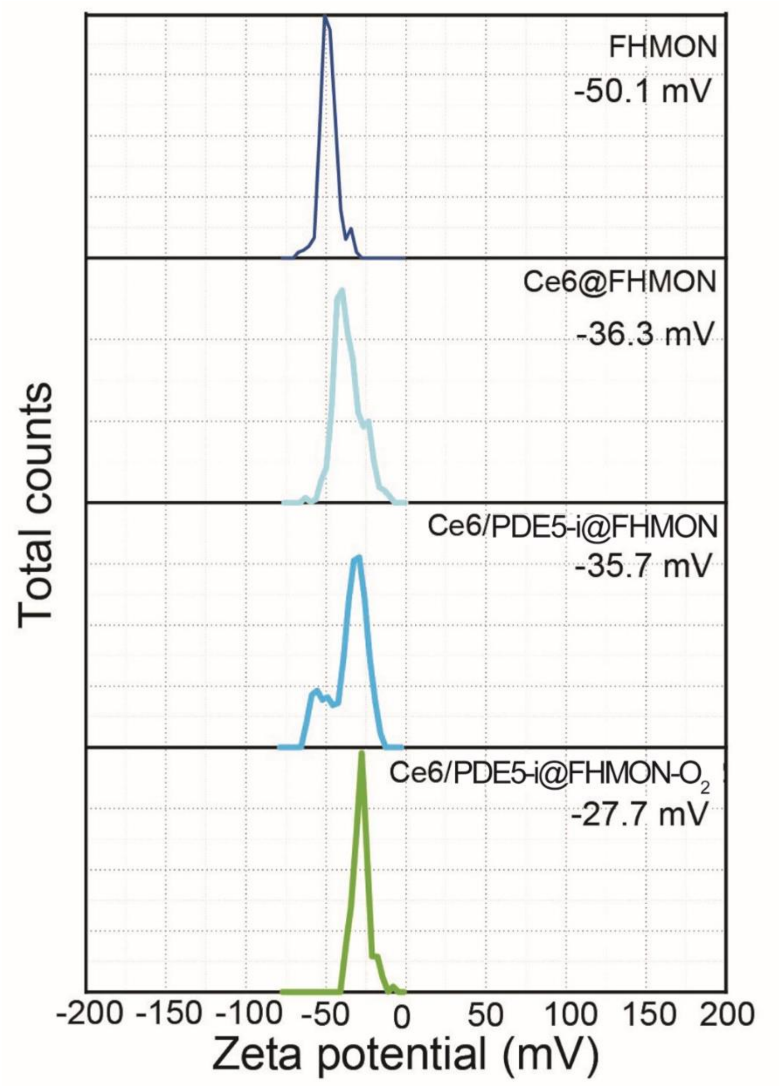

Figure S2 Zeta potential scanning profiles of different samples, *e.g.*, FHMON, Ce6&FHMON, Ce6/PDE5-i@FHMON, and Ce6/PDE5-i@FHMON-O_2, which are determined by dynamic light scattering (DLS).

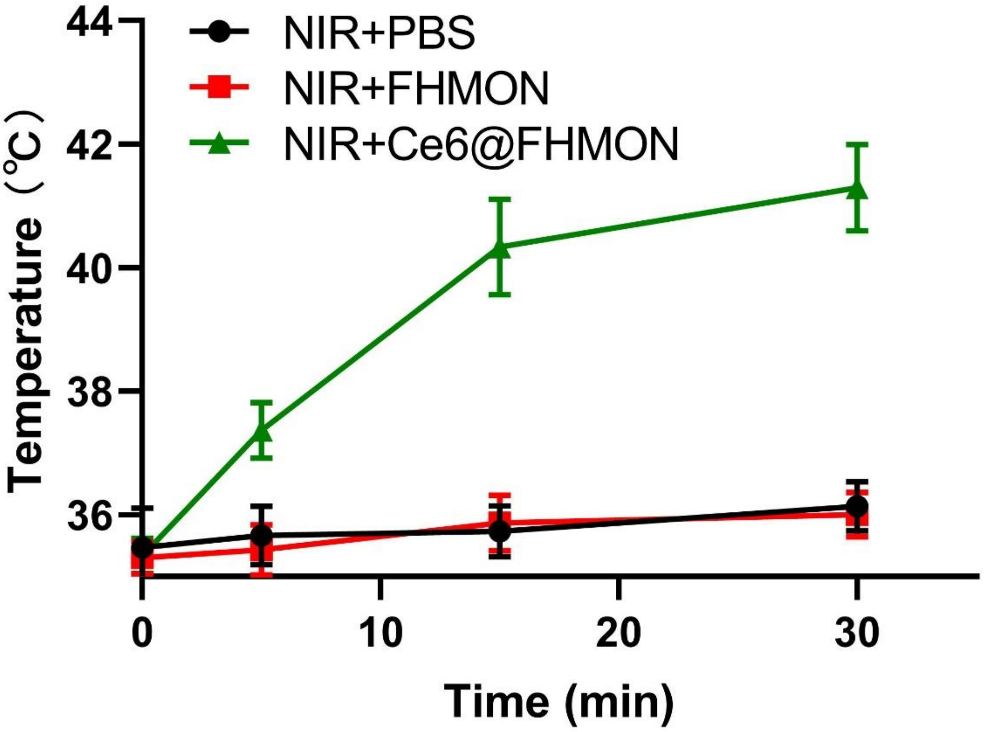

Figure S3 Temperatures of different samples, *e.g.*, NIR+PBS, NIR+FHOMN, and NIR+Ce6@FHMON, which are induced by NIR irradiation. Data are expressed as mean ± standard deviation (SD) (n = 3).

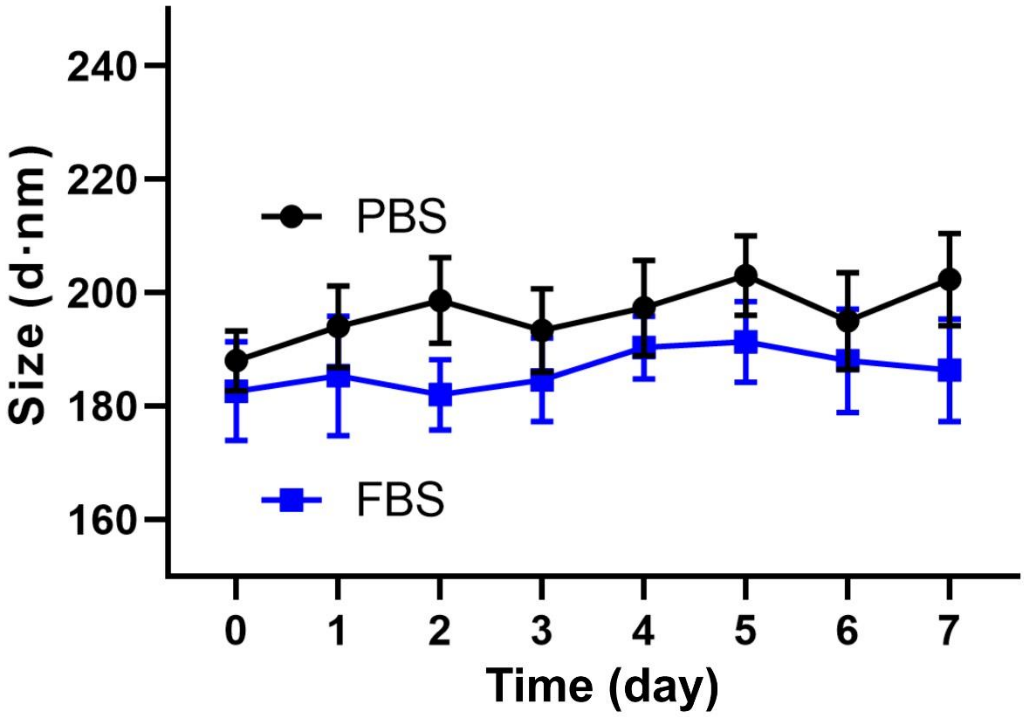

Figure S4 Particle size variations of Ce6/PDE5-i@FHMON-O_2 in PBS and fetal bovine serum (FBS) in 7 days. Data are expressed as mean ± standard deviation (SD) (n = 3).

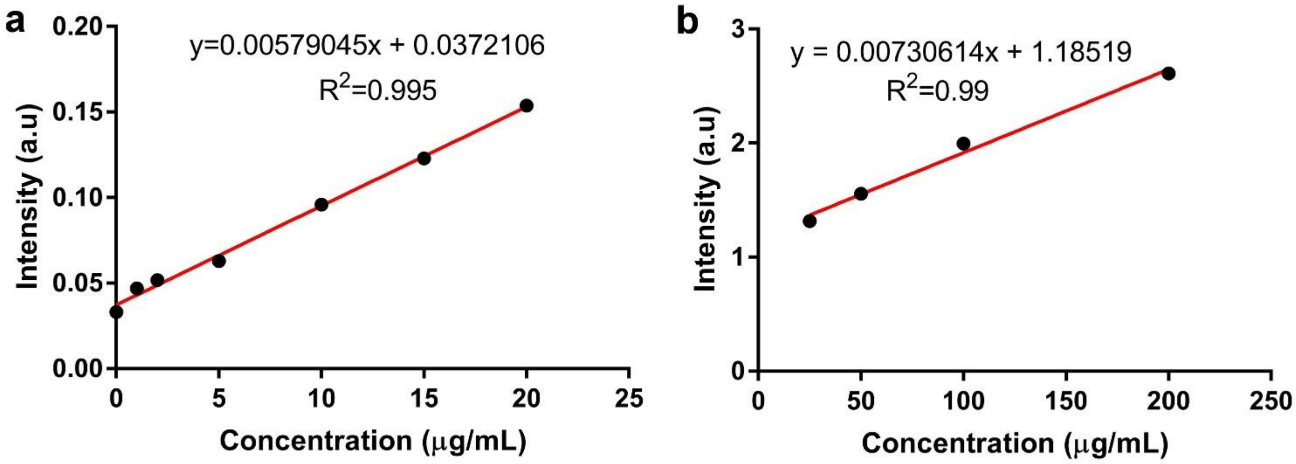

Figure S5 (a) The standard curve of Ce6 concentration relating to its characteristic peak intensity at 506 nm in UV-vis spectra; (b) The standard curve of PDE5-i concentration relating to its characteristic peak intensity at 290 nm in UV-vis spectra.

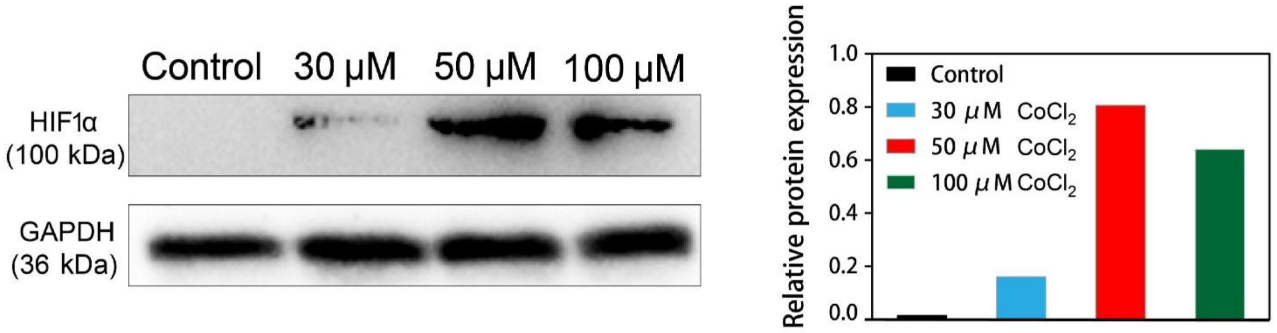

Figure S6 Western blot bands and semi-quantitative analysis of hypoxia-related proteins (*i.e.*, HIF1α) in MCF-7 cells after incubating with $CoCl_2$ with varied concentrations (*e.g.*, 0 μM (Control), 30 μM, 50 μM and 100 μM).

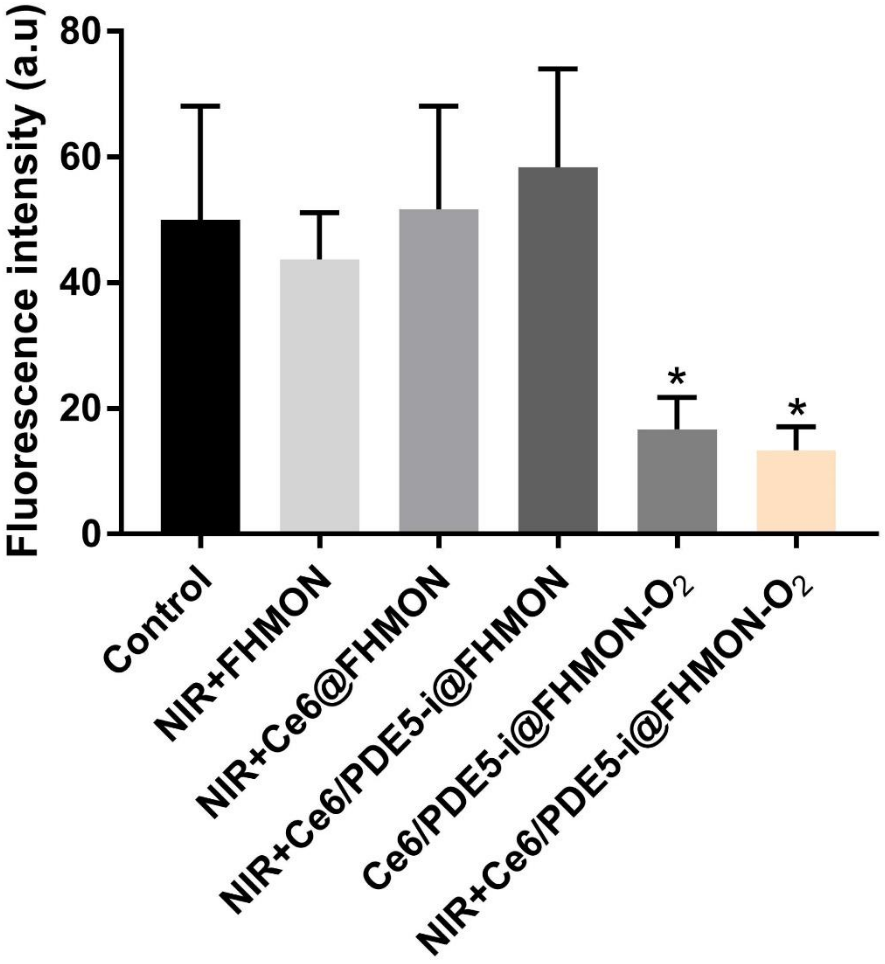

Figure S7 Semi-quantitative fluorescence intensity of hypoxia in hypoxic MCF-7 cells (50 µM, $CoCl_2$) after different treatments, *e.g.*, Control, NIR+FHMON, NIR+Ce6@FHMON, NIR+Ce6/PDE5-i@FHMON, Ce6/PDE5-i@FHMON-O_2 and NIR+Ce6/PDE5-i@FHMON-O_2. Data are expressed as mean ± SD (n = 3). Statistical analyses were performed using a Student's *t*-test, and $^*P < 0.05$.

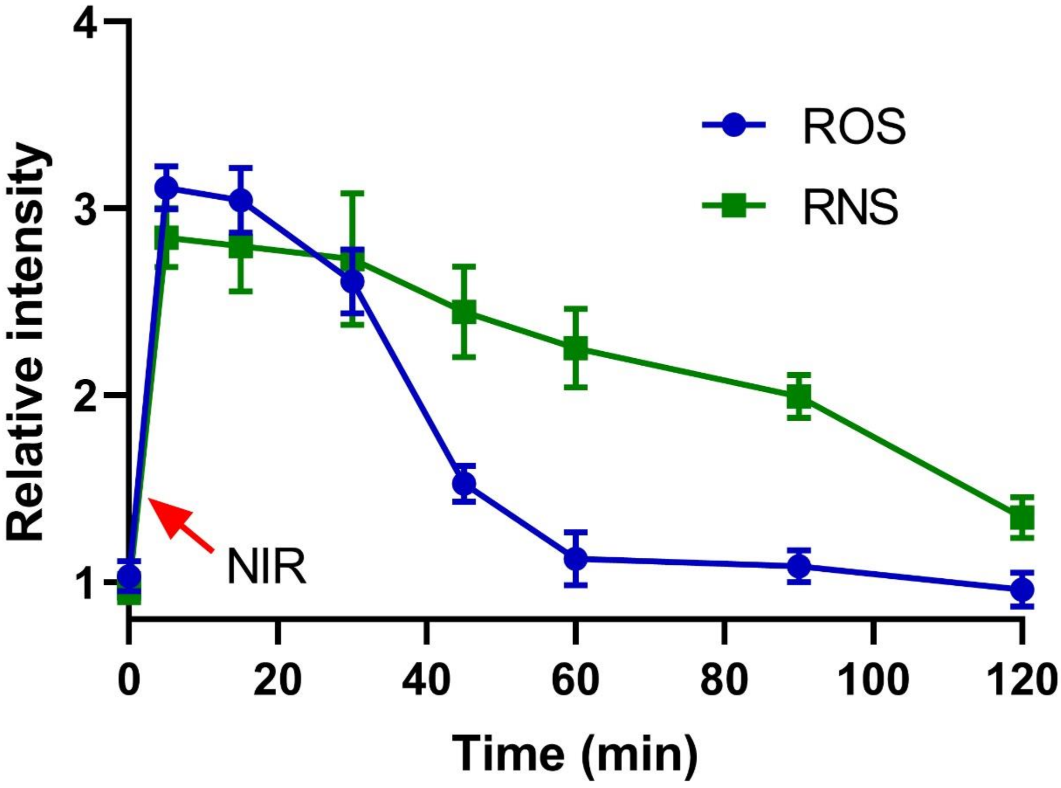

Figure S8 Time depemdent variation profiles of ROS and RNS levels in MCF-7 cells after treatment with NIR+Ce6/PDE5-i@FHMON-O_2. Data are expressed as mean ± SD (n = 3).

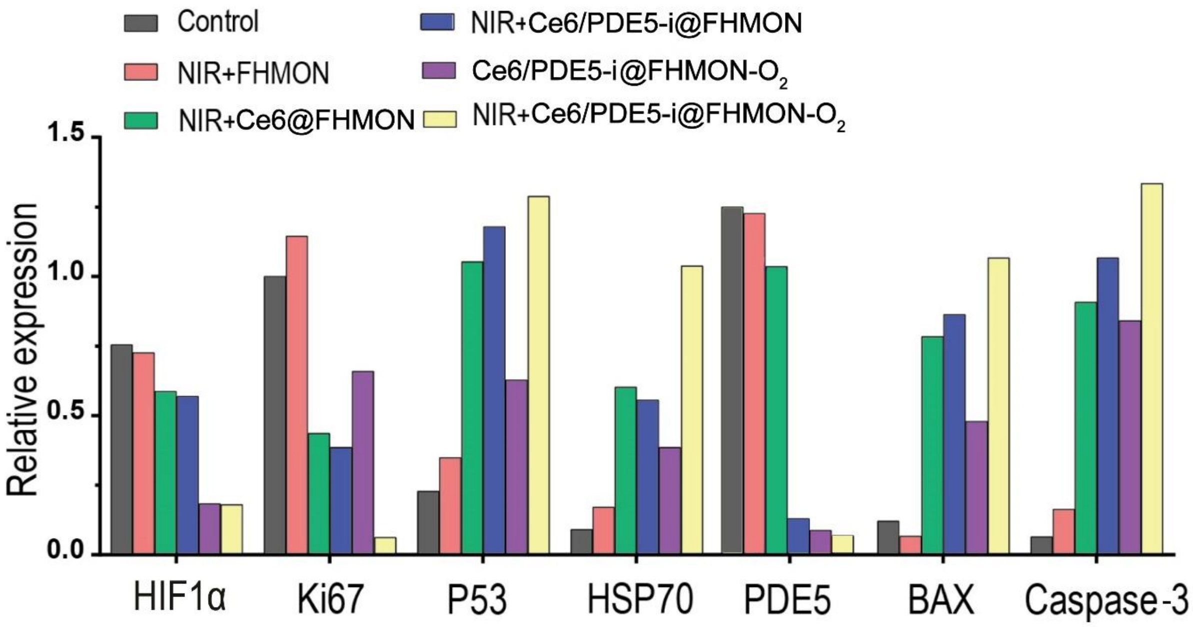

Figure S9 Semi-quantitative expression levels of different proteins (BAX, Ki67, HSP70, Caspase-3, P53, PDE5 and HIF1α) in hypoxic MCF-7 cells (50 µM $CoCl_2$) *via* WB analysis after different treatments, *e.g.*, Control, NIR+FHMON, NIR+Ce6@FHMON, NIR+Ce6/PDE5-i@FHMON, Ce6/PDE5-i@FHMON-O_2 and NIR+Ce6/PDE5-i@FHMON-O_2.

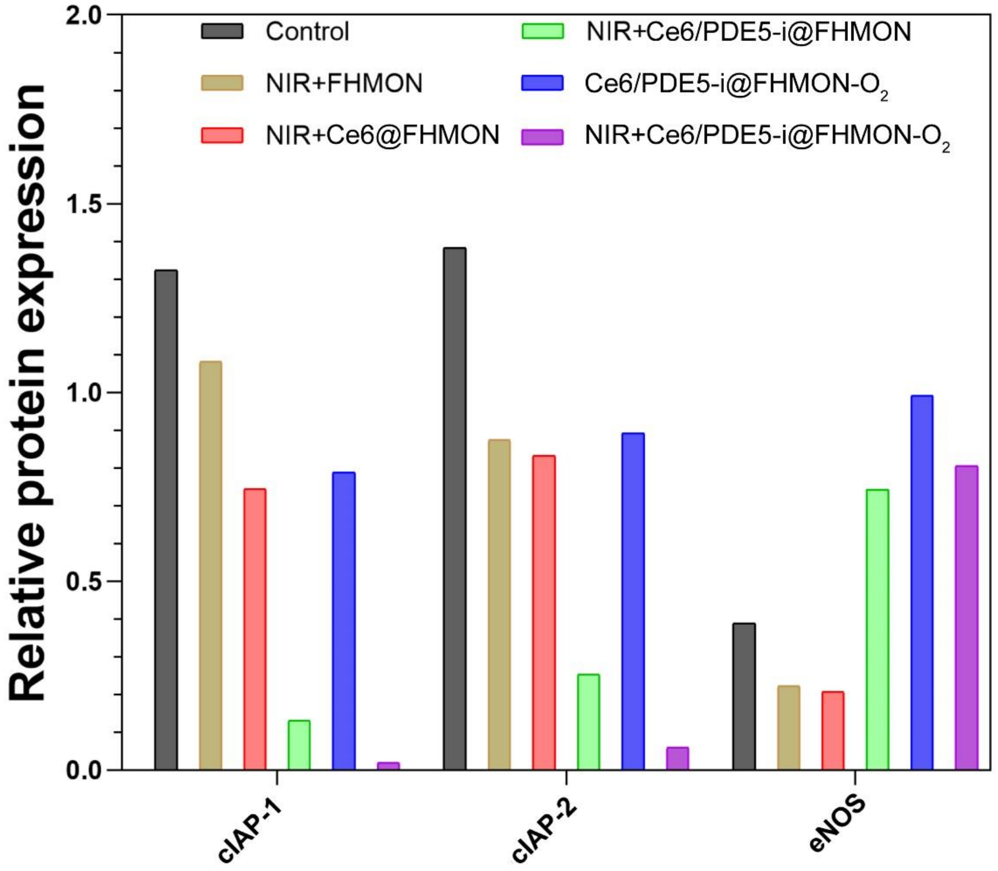

Figure S10 Semi-quantitative expression levels of different proteins (cIAP-1, cIAP-2 and eNOS) in hypoxic MCF-7 cells (50 µM, $CoCl_2$) *via* WB analysis after different treatments, *e.g.*, Control, NIR+FHMON, NIR+Ce6@FHMON, NIR+Ce6/PDE5-i@FHMON, Ce6/PDE5-i@FHMON-O_2 and NIR+Ce6/PDE5-i@FHMON-O_2.

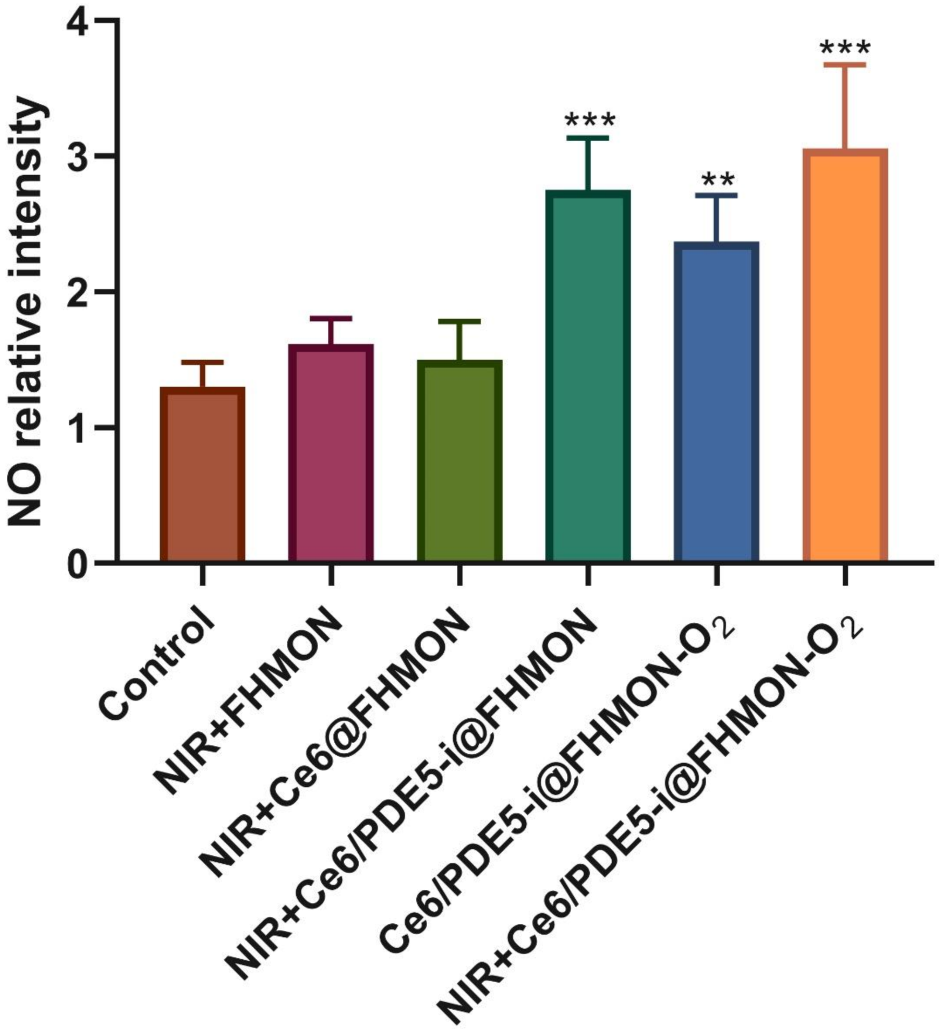

Figure S11 NO production in hypoxic MCF-7 cells after different treatments *via* griess kit assay, *e.g.*, Control, NIR+FHMON, NIR+Ce6@FHMON, NIR+Ce6/PDE5-i@FHMON, Ce6/PDE5-i@FHMON-O_2 and NIR+Ce6/PDE5-i@FHMON-O_2. Data are expressed as mean ± SD (n = 3). Statistical analyses were performed using a Student's *t*-test, and $^{**}P < 0.01$ and $^{***}P < 0.001$.

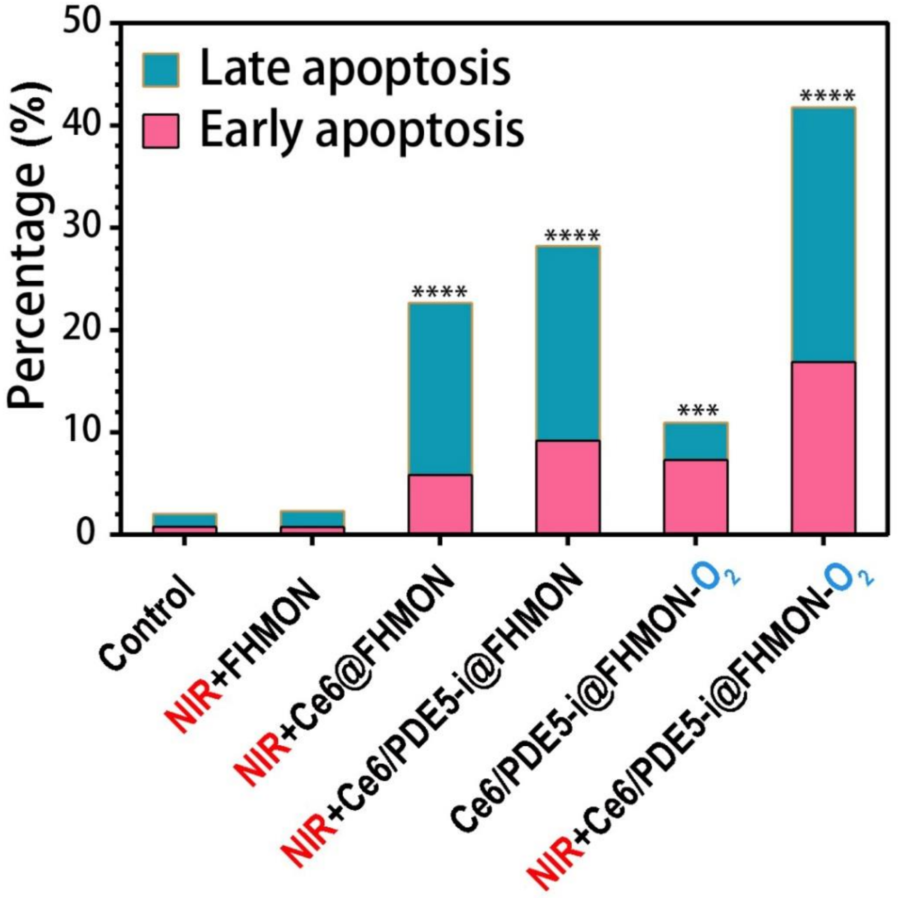

Figure S12 Quantitative apoptosis percentages of hypoxic MCF-7 cells (50 µM, $CoCl_2$) *via* FCM after different treatments, *e.g.*, Control, NIR+FHMON, NIR+Ce6@FHMON, NIR+Ce6/PDE5-i@FHMON, Ce6/PDE5-i@FHMON-O_2 and NIR+Ce6/PDE5-i@FHMON-O_2, where late and early apoptosis were classified. Note, "***" and "****" represent $P < 0.001$ and 0.0001, respectively.

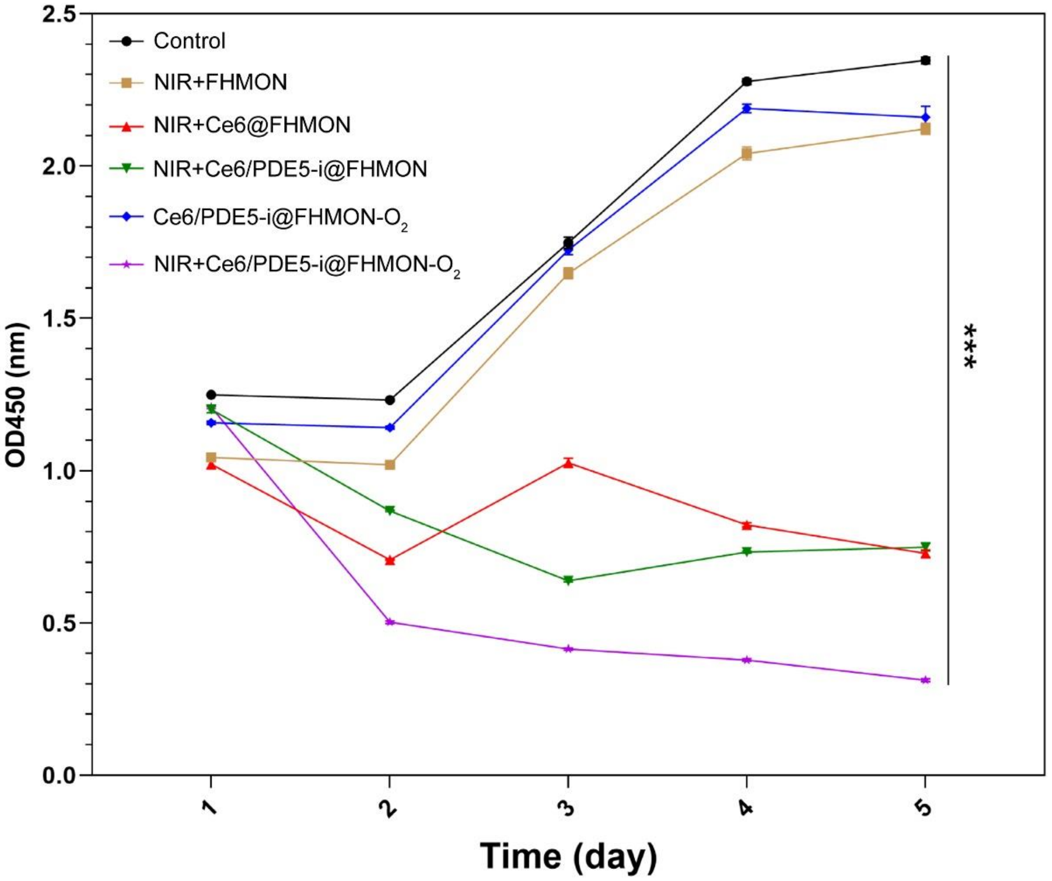

Figure S13 CCK8-determined cell viability of hypoxic MCF-7 cells (50 μM $CoCl_2$) after different treatments, *e.g.*, Control, NIR+FHMON, NIR+Ce6@FHMON, NIR+Ce6/PDE5-i@FHMON, Ce6/PDE5-i@FHMON-O_2 and NIR+Ce6/PDE5-i@FHMON-O_2. Data are expressed as mean ± SD (n = 3). Statistical analyses were performed using a Student's *t*-test, and $^{***}P < 0.001$.

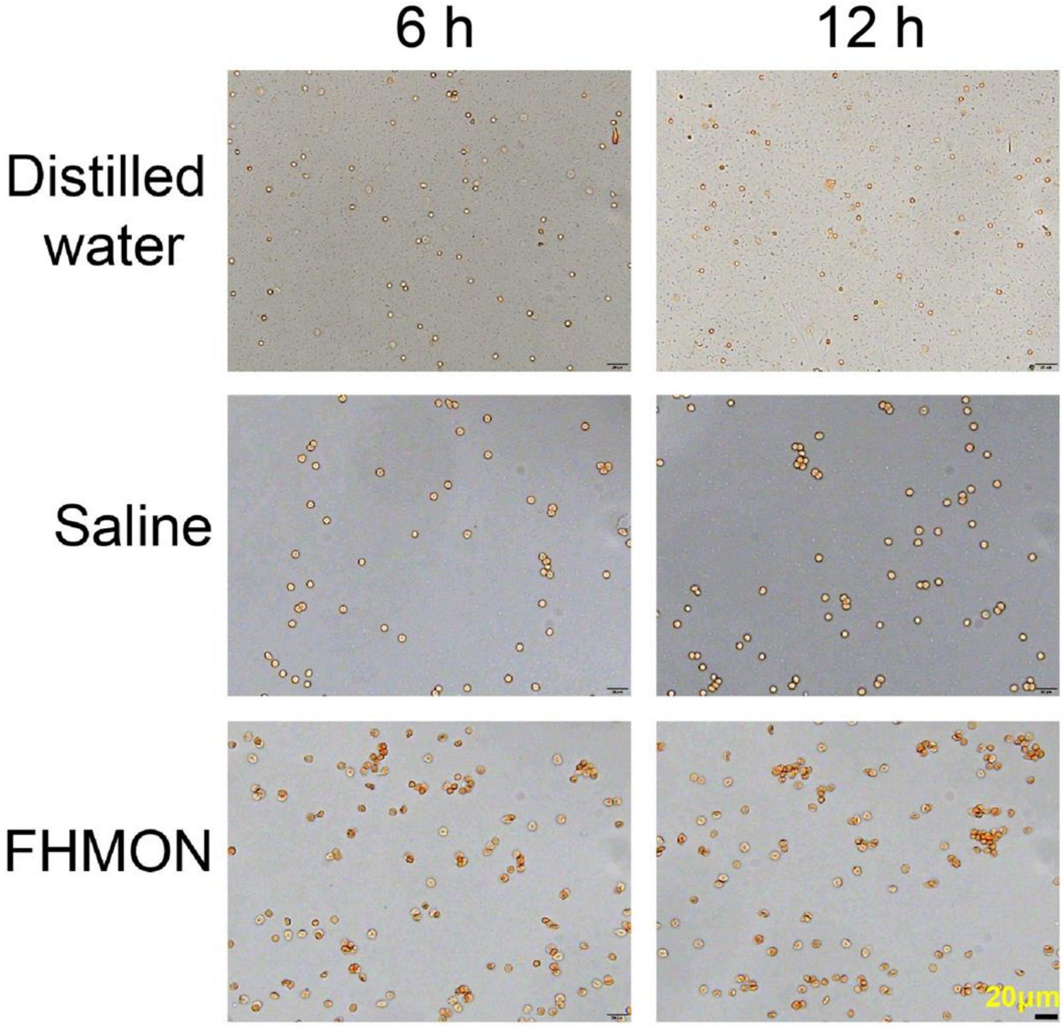

Figure S14 Optical microscopic images of blood cells after incubations with different samples (*e.g.*, distilled water, saline and FHMON) for assessing the hemolysis. Scale bar = 20 μm.

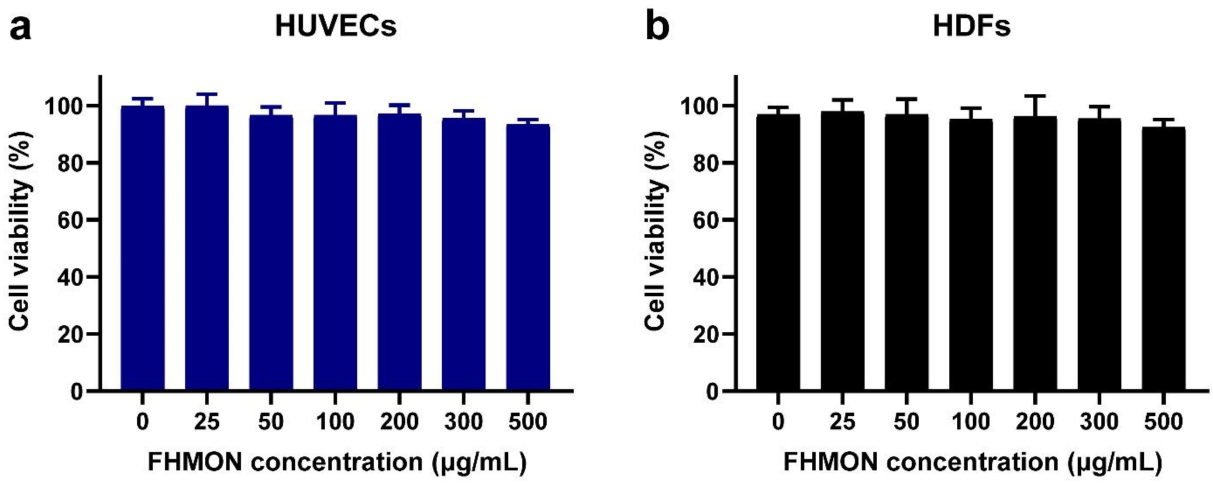

Figure S15 Cell viability of HUVEC (a) and HDF (b) cells after incubations with FHMON with varied concentrations (*e.g.*, 0, 25, 50, 100, 200, 300 and 500 μg /mL) for assessing the cytotoxicity. Data are expressed as mean ± SD (n = 6).

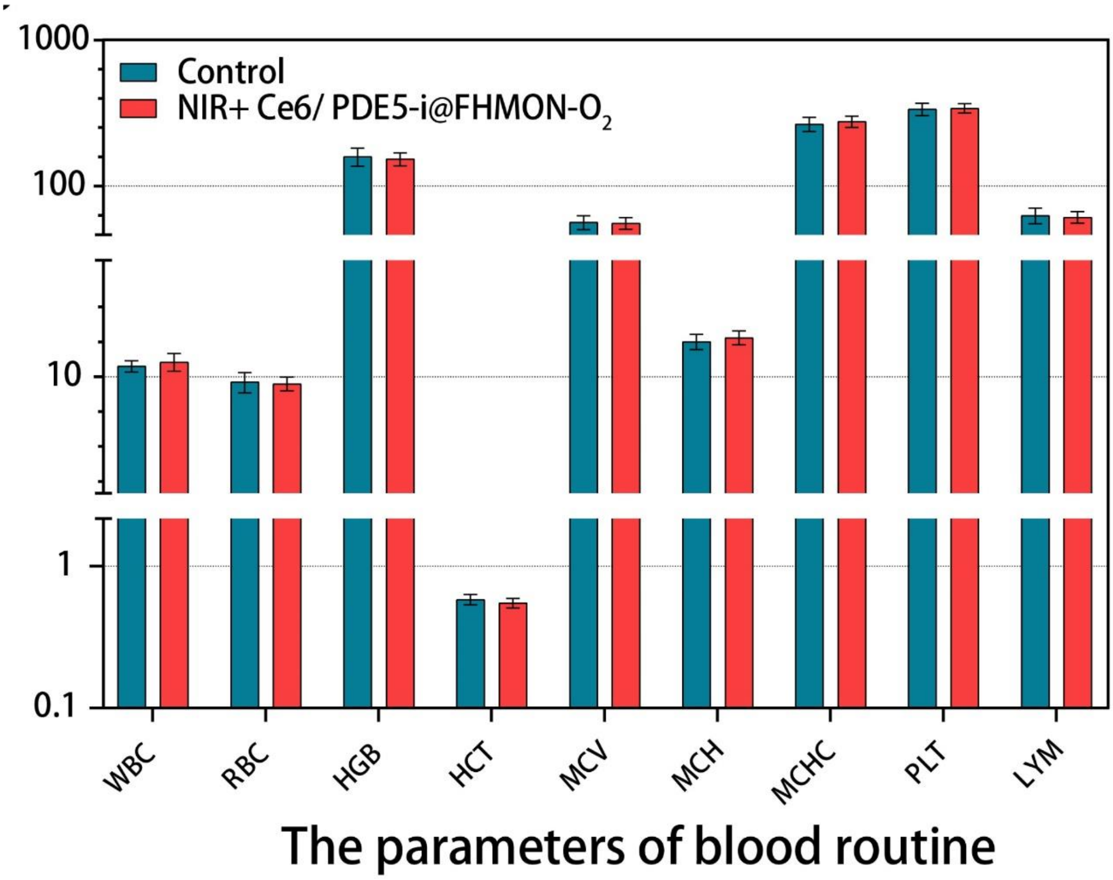

Figure S16 Routine blood indexes of mice in two groups, *i.e.*, Control and NIR+Ce6/PDE5-i@FHMON-O_2, where WBC-white blood cells, RBC- red blood cells, HGB-hemoglobin, HCT- hematocrit, MCV-mean corpuscular volume, MCH-mean corpuscular hemoglobin, MCHC- mean corpuscular hemoglobin concentration, PLT- platelets and LYM-lymphocytes. Data are expressed as mean ± SD (n = 5).

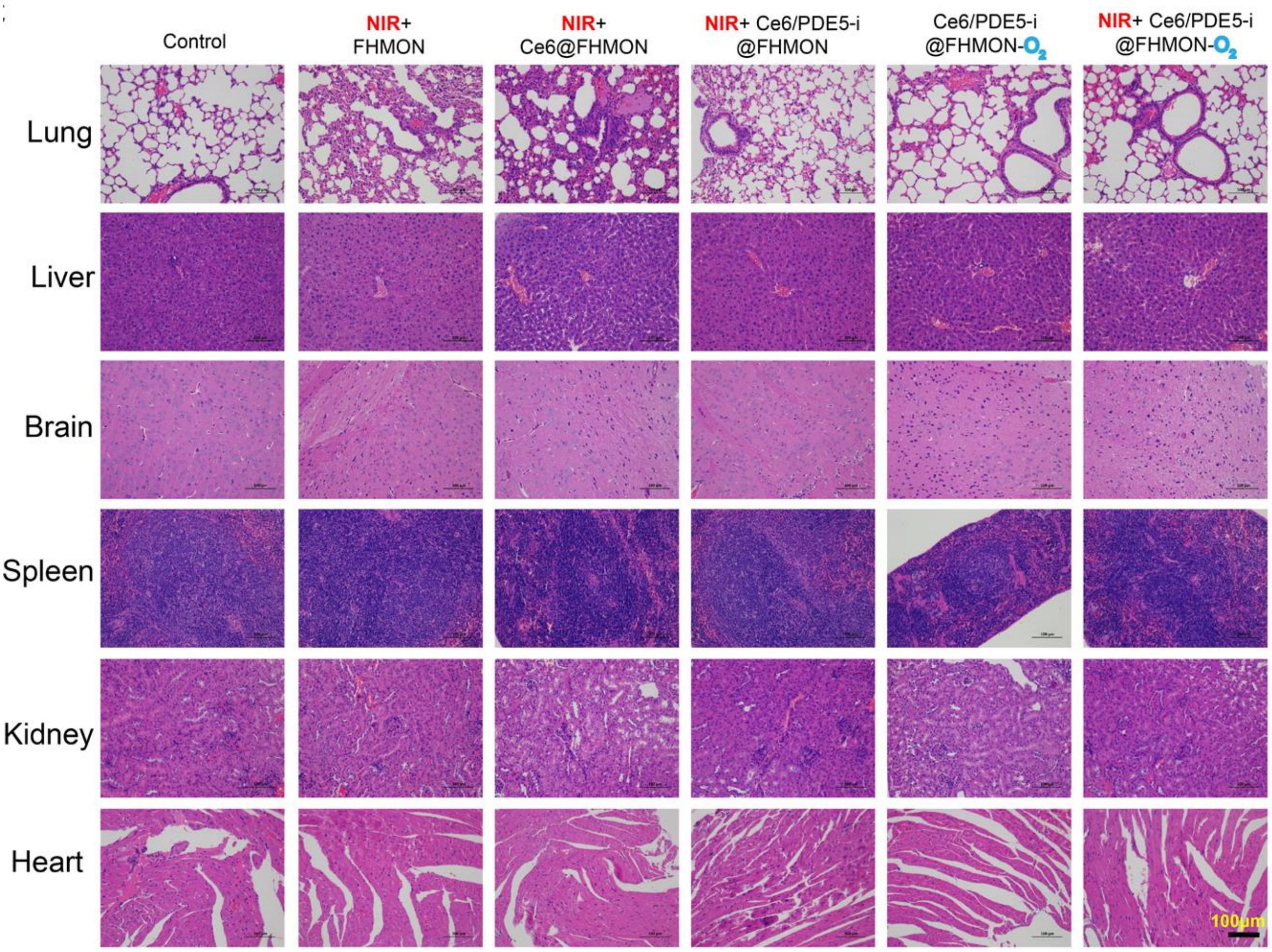

Figure S17 H&E microscopic images of normal tissue slices (*e.g.*, heart, liver, spleen, brain, kidney and lung) harvested from hypoxic MCF-7 tumor-bearing mice that experienced different treatments.

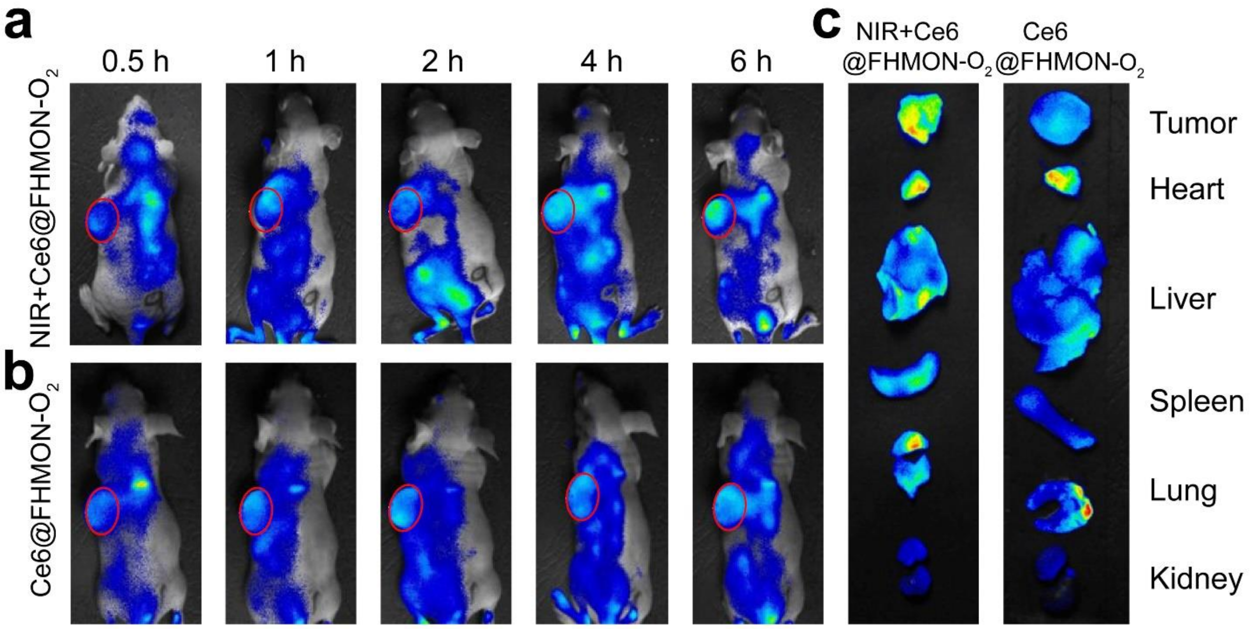

Figure S18 (a,b) Time-dependent *in vivo* fluorescence images of MCF-7 hypoxic tumor-bearing nude mice that experienced treatments with NIR+Ce6@FHMON-O_2 (a) and Ce6@FHMON-O_2 alone (b). (c) *Ex vivo* fluorescence images of tumor and main organs (*e.g.*, heart, liver, spleen, brain, kidney and lung) that experienced treatments with NIR+Ce6@FHMON-O_2 and Ce6@FHMON-O_2 after 6 h. The excitation wavelength was set as 660 nm in terms of Ce6. Red circles indicate the tumor.

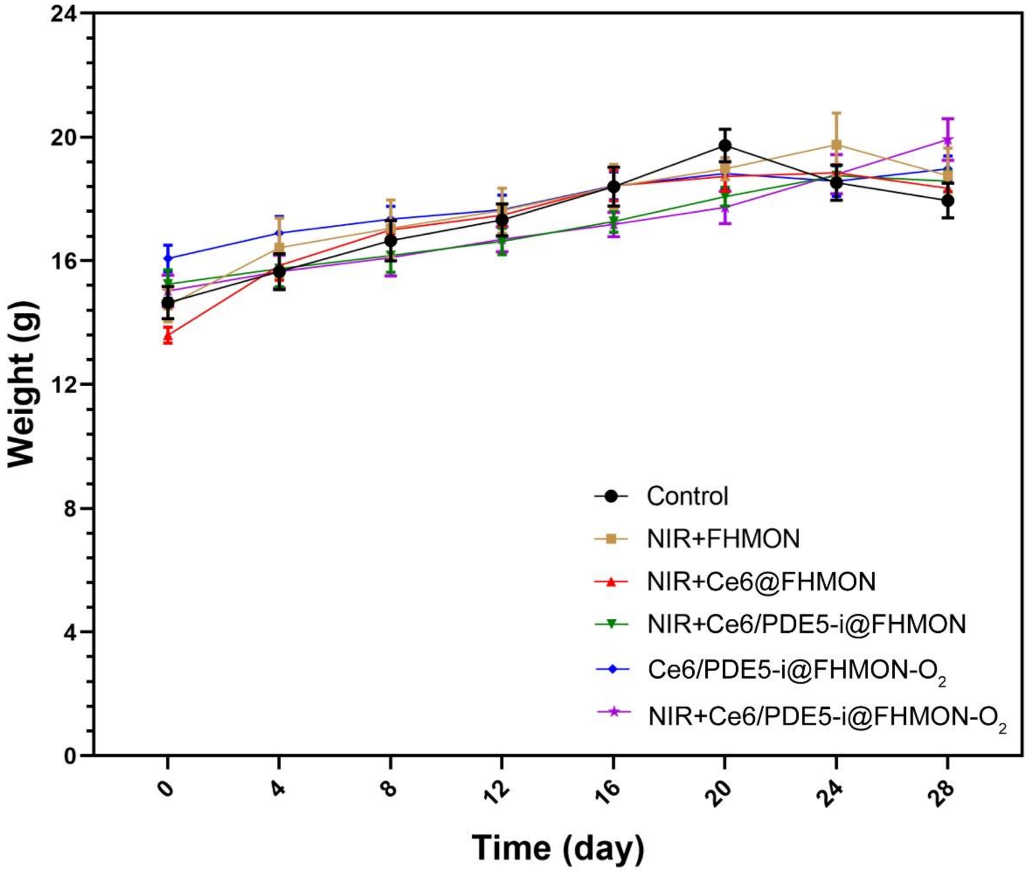

Figure S19 Time-dependent weight variation profiles of MCF-7 tumors-bearing nude mice that experienced different treatments, *e.g.*, Control, NIR+FHMON, NIR+Ce6@FHMON, NIR+Ce6/PDE5-i@FHMON, Ce6/PDE5-i@FHMON-O_2 and NIR+Ce6/PDE5-i@FHMON-O_2. Data are expressed as mean ± SD (n = 4).

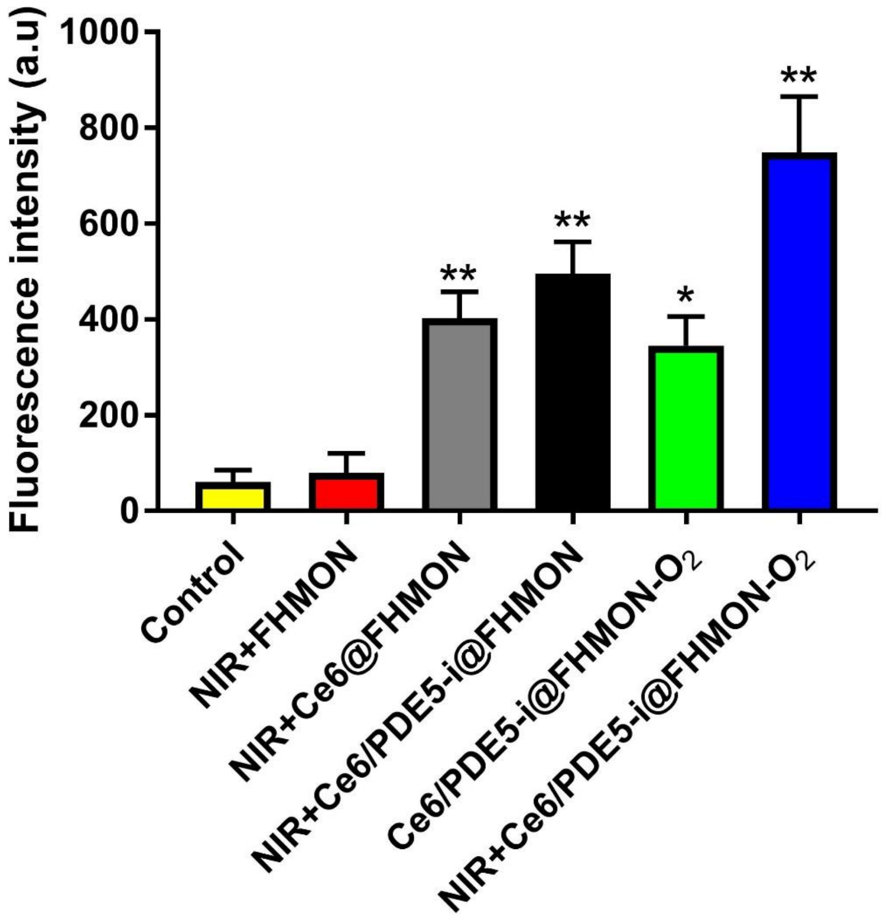

Figure S20 Semi-quantitative fluorescence intensity of apoptotic MCF-7 cells in isolated MCF-7 tumor slices after TUNEL immunofluorescence staining post-different treatments, *e.g.*, Control, NIR+FHMON, NIR+Ce6@FHMON, NIR+Ce6/PDE5-i@FHMON, Ce6/PDE5-i@FHMON-O_2 and NIR+Ce6/PDE5-i@FHMON-O_2. Data are expressed as mean ± SD (n = 3). Statistical analyses were performed using a Student's *t*-test, and $*P < 0.05$ and $**P < 0.01$.

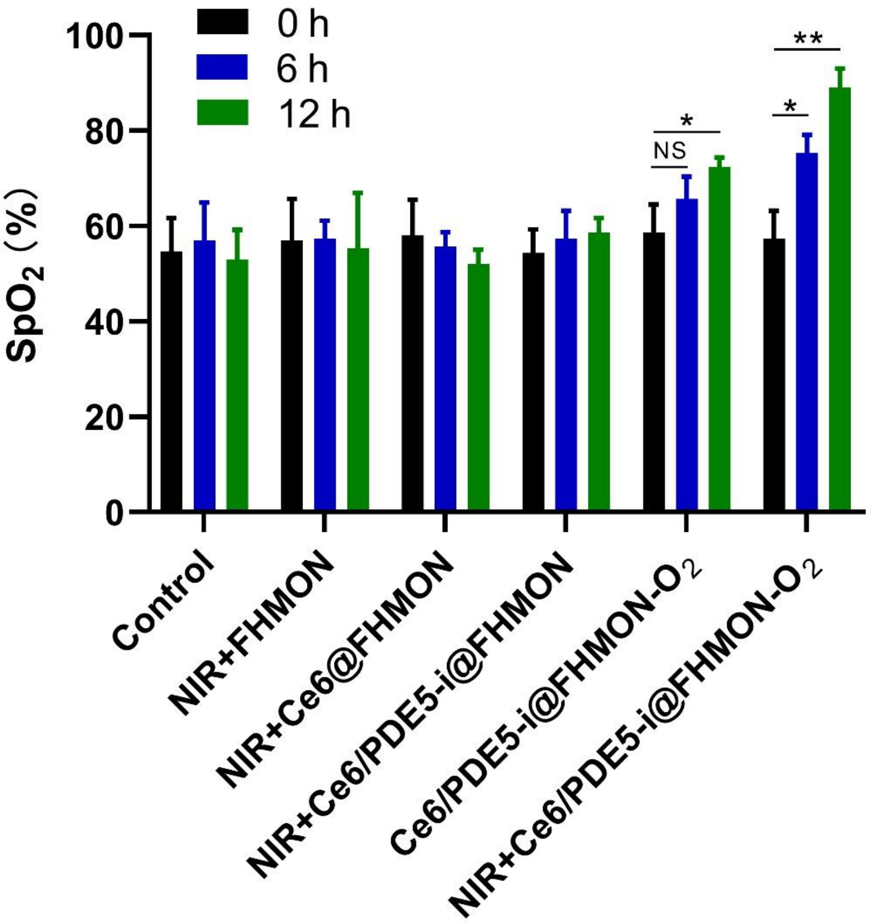

Figure S21 Time-dependent SpO_2 contents at the site of MCF-7 solid tumor implanted in nude mice that experienced different treatments with Control, NIR+FHMON, NIR+Ce6@FHMON, NIR+Ce6/PDE5-i@FHMON, Ce6/PDE5-i@FHMON-O_2 and NIR+Ce6/PDE5-i@FHMON-O_2, respectively. Data are expressed as mean ± SD (n = 3). Statistical analyses were performed using a Student's *t*-test, and $*P < 0.05$ and $**P < 0.01$. NS, not significant.

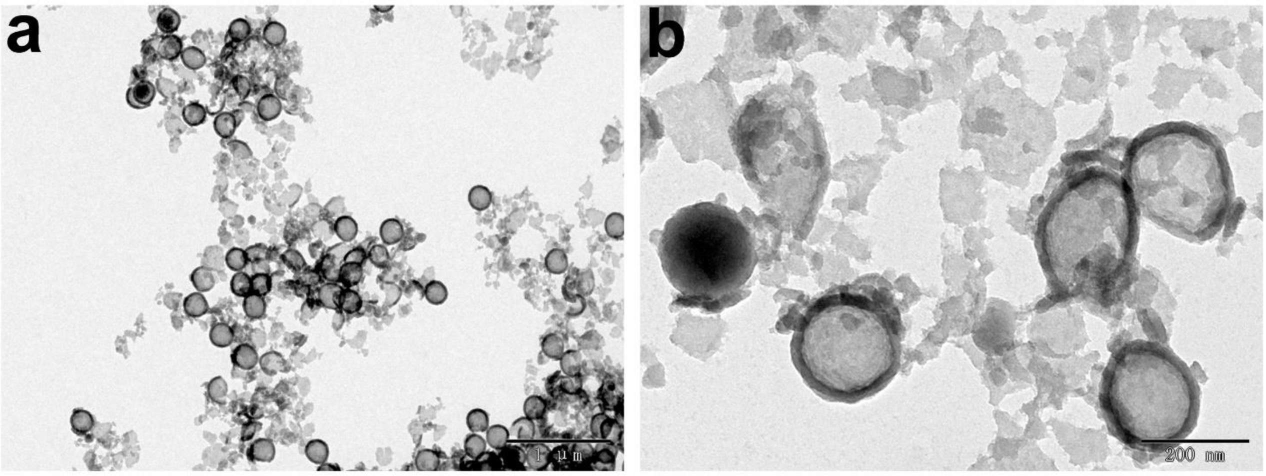

Figure S22 TEM images of FHMON carriers after 26 days post-synthesis, and the carriers were stored at 4 °C in refrigerator. Evident debris and shell vanishment are observed, indicating the degradation.
